# IFNL3 reduces replication of CIRDC-associated viruses in canine airway epithelial cells

**DOI:** 10.1016/j.virusres.2026.199742

**Published:** 2026-05-03

**Authors:** Swati Sharma, Glorián Berríos-Vázquez, Kennedy Baldwin, Roger Maes, Gisela Soboll Hussey

**Affiliations:** a College of Veterinary Medicine, Department of Pathobiology and Diagnostic Investigation, Michigan State University, USA; b Veterinary Diagnostic Laboratory, Michigan State University, USA; c College of Natural Science, Department of Microbiology and Genetics and Immunology, Michigan State University, USA

**Keywords:** Kennel cough, Antiviral, Canine respiratory epithelial cells, Prevention, mRNA, Viral titer, Interferons

## Abstract

Canine infectious respiratory disease complex (CIRDC) causes contagious respiratory disease in dogs and encompasses several etiologic agents. Viral pathogens associated with CIRDC include canine adenovirus type 2 (CAV-2), canine distemper virus (CDV), canine herpesvirus-1 (CaHV-1) and canine influenza virus (CIV). Vaccine availability and efficacy vary and there are currently no antivirals available for treatment. Interferons (IFNs) are at the frontline of the immune defense against many viral infections and interferon lambda (IFNL) has shown to be a potent antiviral. We hypothesized that canine interferon lambda-3 (IFNL3) treatment of canine respiratory epithelial cells (ALI-CRECs) will reduce replication of kennel cough viruses.

ALI-CRECs were isolated, cultured and characterized morphologically and immunologically prior to infection with CDV, CaHV-1, CIV and CAV-2. The value of canine IFNL3 treatment for reducing viral titers as well as induction of interferon responses were evaluated for each virus.

Treatment of ALI-CRECs with IFNL3 induced the expression of interferon stimulated genes. In addition, prophylactic IFNL3 administration resulted in a reduction in viral DNA with CT value changes of up to 13 intracellularly and up to 9 extracellularly. In contrast, for CDV and CaHV-1 a reduction in viral RNA/DNA was observed only intracellularly with CT value changes of up to 11 and up to 4 respectively. Prophylactic IFNL3 treatment also significantly reduced viral titers 1.2 to 3 log-fold for CAV-2, 0.9–2.5 log-fold for CDV, and 1.1–1.7 log-fold for CaHV-1. No effect of IFNL3 was observed for CIV RNA and CIV viral titers. Coinciding with a reduction in viral replication, interferon stimulated genes were differentially expressed in interferon treated and infected cells when compared with controls.

In conclusion, ALI-CRECs have shown to be excellent systems to study respiratory viruses and evaluate the potential antiviral effect of IFNL3. However further in-depth mechanistic studies are needed to fully understand how IFNL3 stimulates/modulates local immune responses to induce antiviral effects. Moreover, while this study highlights the potential antiviral efficacy of IFNL3 for several canine respiratory viruses, determination of safety, effective doses *in vivo* as well as therapeutic potential will have to be evaluated in dogs.

## Highlights

• Several viruses contribute to the canine infectious respiratory disease complex.
• Canine respiratory epithelial cells (CRECs) are excellent tools tp study infections disease.
• CRECs support viral replication of CAV-2, CaHV-1, CDV and CIV(H3N2).
• IFNL3 treatment of CRECs induced viperin, MXI and IRF7 mRNA expression.
• IFNL3 treatment reduced CAV-2, CaHV-1 and CDV replication in CRECs.

## Graphical abstract

**Figure:**
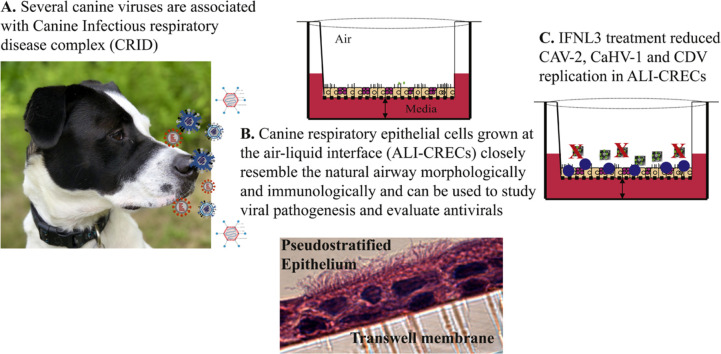

## 1 Introduction

The canine infectious respiratory disease complex (CIRDC), or “kennel cough,” is a multifactorial disease. Contributing factors are infection with several different pathogens, the immune status of dogs, and environmental factors. The predominant clinical sign is acute respiratory disease, and it is common in dogs housed in crowded environments i.e. shelters and kennels (Buonavoglia and Martella, 2007; Day et al., 2020). Clinical symptoms observed include fever, dry “honking cough” and oculo-nasal discharge (Ford and Vaden, 1998). Common pathogens that contribute to CIRDC include Bordetella bronchiseptica, canine adenovirus-2 (CAV-2), canine influenza virus (CIV) H3N2, canine distemper virus (CDV), and canine herpesvirus-1 (CaHV-1) (Mitchell and Brownlie, 2015). The most common viral causes of kennel cough are CAV-2, CDV, CaHV-1, and CIV (Reagan and Sykes, 2020). These viruses initially infect the epithelial cells of the upper respiratory tract and cause inflammation, leading to destruction of the upper respiratory epithelium. CIRDC-associated infection with CAV-2 and CIV is self-limiting, but CDV can lead to neurological manifestations as the virus spreads from the respiratory tract to the lymphoid tissues, and from there to epithelial cells of different organs, including the central nervous system (CNS). CDV viremia is clinically characterized by fever, gastrointestinal or dermatological symptoms and nasal discharge (Elia et al., 2015). Infections caused by CaHV-1 predominantly affect the respiratory tract and the genital tract of the dogs. In addition, CaHV-1 establishes latency in the sensory ganglia. During stress or immune suppression CaHV-1 can then reactivate and lead to renewed disease (Kumar et al., 2015). Due to genetic variation of the pathogens and immunomodulation currently available vaccines are often only effective for a short duration and protection is incomplete. In addition, vaccines can decrease the severity of clinical symptoms, but they are not preventing disease completely (Edinboro et al., 2004).

Because the pathogens causing CIRDC infect via the respiratory mucosa, stimulation of mucosal immunity is likely to improve local mucosal protection as well as aid with induction and shaping of appropriate systemic and adaptive responses for protection from CIRDC (Avadhanula et al., 2006; Priestnall et al., 2009). Early innate immunity acts as a primary defense mechanism and requires no prior exposure when encountering a pathogen for the first time (Marshall et al., 2018). For respiratory infections, mucosal epithelial cells are central for recognizing pathogens via pattern recognition receptors and activation of transcription factors such as interferon regulatory factors 3 and 7 (IRF-7 & IRF-3), and nuclear factor-kappa beta (NF-kb). These transcription factors then further activate interferon responses (Holmgren and Czerkinsky, 2005; Kim and Jang, 2014), which are known to be critical in the antiviral defense (Kohara et al., 2012). Type I (IFN-α and IFN-β) and type III interferons (interferon lambda -1, interferon lambda-2, and interferon lambda-3) are important for the early antiviral response at the mucosa, while type II interferons (interferon-γ) are considered part of the adaptive immune response (Lazear et al., 2015).Whereas type I interferon receptors are present ubiquitously in most cell types, type III interferon receptors (IL10Rß and IFNLR1) are mainly expressed on epithelial cells that line the mucosal surface in addition to some immune cells and consequently, fewer side effects are observed compared to type I interferon. Additionally, unlike type I interferons, IFNL3 does not trigger CXCR3 ligand–mediated inflammatory responses, while still demonstrating antiviral activity across various veterinary species, including dogs (Ye et al., 2019). Moreover, IFNL1 expressed in E.coli has shown to inhibit canine influenza infection and induce interferon stimulated genes in vitro in a dose dependent manner with no inflammatory side effects. Additionally, adenoviral vector based canine interferon lambda (Ad-caIFNλ3) enhanced immunity to canine influenza in vitro*.* Together, both studies suggest that Type III interferon maybe effective antivirals in dogs (Ichihashi et al., 2013; Kim et al., 2021). Similarly, type III IFN has shown to be an important component of the first line of defense against other viral pathogens that infect via the respiratory tract (Ye et al., 2019). In cattle, use of recombinant IFNL3 inhibited the replication of foot and mouth viral disease in cattle (Perez-Martin et al., 2012). In ferrets it has been shown that the use of IFLN3 as an immunoadjuvant enhanced immunity against influenza B virus (Rowe et al., 2024). Miller et al. showed that pegylated IFN-λ1 (Peg-IFN-λ1) used to treat hepatitis C virus infection in animals during a phase 1 clinical trial caused fewer toxic effects and generated better antiviral effects compared to Peg-IFN-α at equal concentrations (Miller et al., 2009).

Because there is evidence for the potential use of IFNL3 as an antiviral in several species including dogs, we hypothesized that recombinant canine IFNL3 would have antiviral potential for respiratory viruses involved in CIRDC of dogs. To test our hypothesis in an in vitro system before animal studies, our first objective was the development of a canine in vitro model that mimics the natural airway of dogs. This was based on evidence that respiratory epithelial cells cultured at air-liquid interface (ALI) have shown to closely mimic the in-vivo conditions of the natural airway, both morphologically and immunologically in several species (Cozens et al., 2018; Prytherch et al., 2011; Runft et al., 2023). Moreover, the value of ALI-cultures for infection studies has been shown in horses, cats and humans (Clark et al., 1995; Gultom et al., 2021; Nelli et al., 2016; Runft et al., 2022; Soboll Hussey et al., 2014). Respiratory epithelial cells cultured at air-liquid interface (ALI-cultures) have been previously established and used for studies with SARS-CoV-2, canine distemper virus, and Bordetella bronchiseptica (Anderton et al., 2004; Farber et al., 2022; Shin et al., 2022). However, while a in depth morphological characterization has been completed for the canine system previously (Runft et al., 2023), none of these studies characterized or used these systems for immunological studies. Therefore, the goal of the present study was to: i) Characterize and compare ALI-CRECs with the natural airway epithelium with a focus on expression of immune marker expression; ii) Study the viral growth kinetics of CAV-2, CDV, CaHV-1, and CIV infection in ALI-CRECs: iii) Determine the effect of IFNL3 treatment on the immune response of ALI-CRECs; and iv) Evaluate the potential of IFNL3 treatment for prevention of ALI-CRECs infection with CAV-2, CDV, CaHV-1, and CIV.

## 2 Materials & methods

### 2.1 Animals and tissue collection

Tracheas from total 8 respiratory healthy dogs were collected within 1 hour following euthanasia for unrelated reasons. Dogs were humanely euthanized by sedating them intramuscularly (IM) with 4mg/Kg Butorphanol and 5mg/kg acepromazine, followed by administering 3 to 5 ml propofol and 1mL/10 pound of fatal plus (Pentobarbital) intravenously (IV). Tracheas from each dog were collected aseptically and immediately washed with sterile 1X PBS and transported in Dulbecco’s minimum essential/ Ham’s F-12 (DMEM/F-12) medium (Life technologies, Grand Island, NY, USA) with 1 % penicillin/streptomycin for further processing. At the time point of tissue collection, nasal swabs were also collected from each dog to check the presence of any current respiratory infection. All animal studies were approved by the Institutional Animal Care and Use Committee (IACUC), and all animal experiments were conducted according to IACUC guidelines at Michigan State University, East Lansing, MI USA.

### 2.2 Isolation of primary canine respiratory epithelial cells

Primary canine respiratory epithelial cells (ALI-CRECs) were isolated and cultured from 6 tracheas based on an adjusted protocol previously described for equine cells (Quintana et al., 2011). Briefly, connective tissues and fat were removed from the collected tracheas. Tissue samples were washed twice with fresh DMEM/F12 and 1X PBS and incubated in 1X sterile PBS for 30 min at 4 °C to remove any blood clots. Tracheal tissue was then transferred to digestion medium (calcium and magnesium-free Minimum Essential Medium (MEM), supplemented with 1.4 mg/mL pronase (Roche, Indianapolis, IN, USA), 0.1 mg/mL DNase (Sigma-Aldrich, St. Louis, MO, USA), 100 IU/mL of penicillin, 100 ug/mL of streptomycin (Thermo Fisher Scientific Inc., Waltham, MA, USA), 0.792 ng/mL of fluconazole (Diflucan) and incubated at 4 °C for 72 to 96 h. After incubation,10 % heat inactivated fetal bovine serum (FBS) (Atlanta Biologicals, Norcross, GA, USA) was added to neutralize the enzymatic reaction. Tracheal tissues were then removed from the digestion medium and washed with DMEM/F12 before pelleting epithelial cells and re-suspension in DMEM/F12 medium supplemented with 1X MEM supplemented with non-essential amino acids (Life Technologies, USA), 100 IU/mL of penicillin, 100 ug/mL of streptomycin, 1X Insulin-Transferrin-Selenium (Life Technologies, USA). Resuspended cells were plated on an uncoated cell-culture petri dish (VWR, Radnor, PA, USA) for 3 to 6 h at 37 °C & 5 % CO_2_ to remove fibroblasts. After incubation, the non-adherent cells were collected and resuspended in freezing media supplemented with bronchial epithelial cell growth medium (BEGM) (Lonza Walkersville Inc., Walkersville, MD, USA), 30 % heat inactivated FBS, and 10 % dimethyl sulfoxide (DMSO) (Sigma-Aldrich, St. Louis, MO, USA) for storage in liquid nitrogen.

### 2.3 Culture and morphological characterization of primary canine respiratory epithelial cells (CRECs) at the air-liquid interface (ALI-CRECs)

For culture, approximately 2.5 million CRECs were seeded onto transwell inserts of 12-well transwell plates (Corning, Tewksbury MA, USA) previously coated with Type IV collagen (Sigma-Aldrich, St. Louis, MO, USA) in growth medium #1 (DMEM/F12 supplemented with 10 % FBS, 1X MEM non-essential amino acids, 100 IU/ml penicillin, 100 ug/ml streptomycin and, 1.25 ug of amphotericin B/mL) for 24 h at 37 °C and 5 % CO_2_. After 24 h, growth medium #1 was replaced with growth medium #2 (DMEM/F12 supplemented with 2 % Ultroser-G 100 IU/mL of penicillin, 100 ug/mL of streptomycin, and 1.25 ug of amphotericin B/ml) and cells were cultured at the air-liquid interface. Growth medium #2 was changed at 24-hour intervals until the ALI-CRECs were fully differentiated, at which point they were used for experiments. For morphological characterization of ALI-CRECs, cells were examined microscopically, and differentiation was confirmed using an inverted microscope with Integrated Modulation Mode (IMC) at 100X total magnification (Leica Microsystems Inc. Buffalo Groove, IL, USA). Additionally, differentiated ALI-CRECs were formalin-fixed and paraffin-embedded for hematoxylin and eosin (H&E) staining as previously described (Nelli et al., 2016).

### 2.4 Immunological comparison of CRECs, ALI-CRECs and the natural airway by PCR and qPCR

For characterization of immunological parameters and comparison of ALI-CRECs with the natural airway and freshly isolated CRECs, total RNA was extracted from snap frozen canine tracheal tissues (*n* = 8), freshly isolated CRECs (*n* = 5), and fully differentiated ALI-CRECs (*n* = 6), as previously described for the feline system (Nelli et al., 2016). Briefly, snap frozen tissues were homogenized using tissue rupture hand-held tissue homogenizer in 1 mL of Trizol reagent (Qiagen, USA). For freshly isolated CRECs and ALI-CRECs, 1 mL of Trizol reagent was used to isolate total RNA from 2.5 million CRECs or a transwell insert containing ALI-CRECs after 2 weeks of culture and differentiation. Total RNA from all samples was extracted using a commercially available kit (RNeasy Plus Universal Mini Kit, QIAGEN) according to the manufacturer’s protocol. The RNA concentration was determined using a Nanodrop spectrophotometer (Thermo Fisher). Synthesis of cDNA was completed using the qScript cDNA synthesis kit (Quantbio, USA), and expression of immune markers such as Toll-like receptors (TLRs), cytokines, chemokines, interferons, and other regulatory genes were analyzed by polymerase chain reaction (PCR) and quantitative polymerase chain reaction (qPCR). For PCR, sequence-specific primers were used (Table 1). PCR was performed using GoTaq® G2Hot Start GreenMasterMix (Promega Corporation, Madison, WI, USA) for each gene. For all reactions, a no template control was used as a negative control. Depending on the product size, PCR products were visualized on 1–2 % agarose gels stained with SYBR Safe DNA Gel Stain (Invitrogen, Thermo Scientific, USA) and documented under UV illumination using the Chemi DocTM XRS+ System (BioRad). For quantification and comparison of mRNA levels of each immune marker in tracheal tissue (*n* = 5), freshly isolated CRECs (*n* = 5), and ALI-CRECs (*n* = 5), RNA expression was quantified by qPCR with the ABI7500 Fast Real Time PCR system, using SYBR green master mix, and previously published primers listed in Table 1. The relative fold change was calculated using the 2^−ΔΔCt^ comparative CT method after normalizing the cycle thresholds (CT) with the housekeeping gene GAPDH.

**Table 1:** Primer sequences used to characterize canine immune markers in CRECs, freshly isolated CRECs and ALI-CRECs using PCR and real time PCR (qPCR).

Gene	Forward Primer (5′-3′) for PCR & qPCR	Reverse Primer (5′-3′) for PCR & qPCR	Reference
TLR-1	TCCCAAAGACCTATCCCTGAA	CCATGACCATCTGGCACAC	Mercier E et al., 2012

TLR-2	TCACTTGGGGAAACACCTCT	TCATACGGAGGGCCAGATAG	Silva E et al., 2012
TLR-3	GCGTGAATTTGACTGAACTCC	TCAAGTTCTTCACGCCTCAG	
TLR-4	CAGCATTCCAGTTTGAAGCA	GGAGTTGTCCGGAAAGGAAT	
TLR-5	CTTCGTCTTCTCCCTGAACG	CTGAACGTCTGGTCCTGGAT	

TLR-6	TGGGGAATAGTCATTTCAACATC	TTGGACCTCTGGTGAGTTCTG	Mercier E et al. 2012
TLR-7	ACATCGCTACGTCCACCAG	CACAACGAAAGCATCATAGCA	
TLR-8	CCTGGATTTAAGCGGGAACT	GTACTTGTCACCTTCGGCATT	
TLR-9	ACCCCCTGTCTCTCCTGGT	ACGGTGAGGTTGTTGTACTTGA	
TLR-10	CCTGTTCAAGCAACCTATCCA	ACTGTGGTGGGCGAAGTAAA	
NOD1	CTGAAAATCAACCGGGAACT	GCCGCAGATGAAGATGGT	
NOD2	ACTGCTGTTGGCCTGACTTT	CCGTGATCTGGAGGTTGTG	

CXCR2	TCATCTTTGCTGTCGTGCTC	TGTGGAAGAAGCCCAGAATC	Chiang HC et al. 2012

IFI16	ACTCCAAAAATCCGTGATCT	GGCGTCCATACACCCA	Luff JA et al. 2013

IL1B	TCTCCCACCAGCTCTGTAACAA	GCAGGGCTTCTTCAGCTTCTC	Wang YS et al. 2007
TGF-B1	CAGAATGGCTGTCCTTTGATGTC	AGGCGAAAGCCCTCGACTT	
IL-2	CATCGCACTGACGCTTGTACTT	CCATCTGTTGCTCTGTTTCCTTT	
IL-4	CATCCTCACAGCGAGAAACG	CCTTATCGCTTGTGTTCTTTGGA	
IL-6	TCCTGGTGATGGCTACTGCTT	GACTATTTGAAGTGGCATCATCCTT	
IL-8	CAAGAGCCAGAAAGAAACCAGAAC	AAAGCTGCCAAGAGAGCAACA	
IL-10	CGCTGTCACCGATTTCTTCC	CTGGAGCTTACTAAATGCGCTCT	

CBD1	TCTACTTGCTGCTGCTGCTTCTGT	ACTTCACTGGGCTCAATGGACTTC	Erles and Brownlie, 2010
CBD103	CCTGTCGCGAAGCCTGTTGG	CGCACCGACCGCTCCTTATTCT	

GAPDH	TCCTGCACCACCAACTGCTT	GTCTTCTGGGTGGCAGTGAT	Ichihashi T et al., 2013

### 2.5 Cells and viruses

Viruses used in the present study for infection experiments, were propagated as follows. CDV (ATCC VR-128 strain) was propagated in Vero SLAM cells. For propagating CaHV-1 (ATCC VR-552 strain), CIV H3N2 (USDA NVSL004-IDV strain) and CAV-2 (ATCC VR-800) MDCK cells were used. Both cell lines & viruses were generously provided by the Veterinary Diagnostic Laboratory, Michigan State University, East Lansing, MI USA. Vero SLAM cells or MDCKs were also used for determination of infectious viral titers. For propagating CDV in Vero SLAM, and CaHV-1 and CAV-2 in MDCK, cells were grown in minimum Essential Medium Eagle (Sigma-Aldrich, St. Louis, MO, USA) supplemented with 2.2 g/L sodium bicarbonate, 1ug/ml ciprofloxacin (Sigma-Aldrich, St. Louis, MO, USA), 25ug/ml gentamycin (Sigma-Aldrich, St. Louis, MO, USA), lactalbumin enzymatic hydrolysate 0.05 g/L (Sigma-Aldrich, St. Louis, MO, USA), 1 % glutamax (ThermoFisher Scientific, USA) and 2 % heat inactivated fetal bovine serum (Atlas biologicals, USA). For CIV propagation in MDCK cells, Minimum Essential Medium Eagle (Sigma-Aldrich, St. Louis, MO, USA) supplemented with 2.2 g/L sodium bicarbonate, 1ug/ml ciprofloxacin (Sigma-Aldrich, St. Louis, MO, USA), 25ug/ml gentamycin (Sigma-Aldrich, St. Louis, MO, USA), lactalbumin enzymatic hydrolysate 0.05 g/L (Sigma-Aldrich, St. Louis, MO, USA), 1 % glutamax (ThermoFisher Scientific, USA), 0.5 % bovine serum albumin (Sigma-Aldrich, St. Louis, MO, USA) and 2ug/ml Trypsin, TPCK-Treated (Sigma-Aldrich, St. Louis, MO, USA) was used.

### 2.6 ALI-CRECs infection with kennel cough viruses (CAV-2, CDV, cahv-1, CIV(H3N2))

Two- week- old, differentiated ALI-CRECs were used for all infection experiments and to evaluate IFNL3 treatment. For inoculation with the different viruses, experiments were repeated in cells from 3 dogs. Prior to inoculation plate well and trans well cultures were washed twice with 1X sterile PBS before inoculating them with either canine adenovirus- 2, canine herpesvirus-1, canine influenza virus (H3N2), or canine distemper virus at an MOI of 0.1 based on previous studies conducted by our group in equine and feline respiratory epithelial cells and using feline and equine herpes viruses (Lee et al., 2019; Tai et al., 2016; Zarski et al., 2021) as well as initial experiments showing that ALI-CRECs support infection, show expected morphological changes within 96 hour period and grow to satisfactory endpoint titers (see Section 3.3). Infected cells were incubated at 37 °C and 5 % CO_2_ for 2 h. After 2 h of incubation, the inoculum was removed, and both transwell and plate well were washed twice. Fresh growth medium #2 was added to the plate well, and plates were incubated at 37 C and 5 % CO_2_ until each collection time point. For quantification of viral DNA/RNA and viral infectious end point titer analysis, we harvested the cells and the supernatants at 72 hpi for each virus (CAV-2, CaHV-1, CIV and CDV). Supernatants were collected from the plate well, and cells were collected from the transwell using accumax and resuspended in 1X PBS. Cells and supernatants were stored at -80 °C for molecular and viral titer analysis. Morphological changes were noted and compared to the 1-hour time point microscopically for each virus at 24-hour intervals following infection with the respective viruses. Viral RNA/DNA was isolated using RNeasy Plus Universal Mini Kit and DNeasy Blood and Tissue Kits following the manufacturer’s instruction (Qiagen, Maryland USA). The virus specific PCR was then performed using GoTaq® G2Hot Start GreenMasterMix (Promega Corporation, Madison, WI, USA) with specific primers shown in Table 2. For quantification of the end point titer, TCID50 assays was performed with virus infected cells collected at 72 hpi. Results were calculated using the Reed and Muench method (Reed and Muench, 1938).

**Table 2:** Primer sequences used to amplify the viral genome of CAV-2, CDV, CaHV-1, and CIV using PCR.

Viral PCR	Forward Primer (5′-3′)	Reverse Primer (5′-3′)	Reference
Canine distemper virus	ACAGGATTGCTGAGGACCTAT	CAAGATAACCATGTACGGTGC	Frisk AL et al., 1999
Canine herpesvirus	TGCCGCTTTTATATAGATG	AAGCGTTGTAAAAGTTCGT	Schulze and Baumgärtner, 1998
Canine adenovirus-2	CCCACAGTACAGCAATGACC	TTGACTTGGCTCTGCAAGTT	Dong J et al., 2022
Canine influenza virus (H3N2)	CAAGCACTAATCAAGAACAAAC	TCTGCTGCTTGTCCTGTACCTT	Wang C et al., 2017

### 2.7 Dose determination of interferon lambda 3 (IFNL3)

Recombinant interferon lambda-3 (IFNL3) was provided by Exalt therapeutics with a stock concentration of 1mg/ml. To determine a dose of IFNL3 that would maintain viability of ALI-CRECs and induce expression of interferon induced genes, a preliminary study was performed. In this first set of experiments, two-week old ALI-CRECs from 3 dogs were treated with different concentrations of IFNL3 (0, 10, 100 and 250 ug/mL) before incubation at 37 °C and 5 % CO_2_ for 72 h. In a follow-up second experiment, ALI-CRECs from the same dogs were treated with different concentrations of interferon lambda 3 (0, 10, 100 and 250 ug/mL) and exposed for one hour (transient exposure) at 37 °C and 5 % CO_2._ After a 1-hour incubation, interferon lambda 3 was removed, and cells were washed twice with 1X PBS and incubated for up to 72 h at 37 °C and 5 % CO_2_. Microscopic images of cells were taken at 1, 24, 24, and 72 h before collection of cells. For collection, cells in each set of experiments were washed with 1 X PBS and harvested in accumax and resuspended in 1X PBS and stored at -80 °C for mRNA expression of interferon stimulated genes.

### 2.8 Determination of the effect of IFNL3 treatment on interferon-stimulated gene and cytokine mRNA expression in ALI-CRECs

For determination of mRNA expression of interferon-stimulated genes and other cytokine and chemokines following treatment with IFNL3, fully differentiated ALI-CRECs (*n* = 6) were treated with 100ug/ml of IFNL3 or media alone for one hour. After removal of the treatment inoculum and addition of media, cells were incubated until the respective collection time points Cells and supernatants were collected at 1 h post treatment (hpt), 24 hpt, 48 hpt, and 72 hpt. RNA was isolated from the cell suspensions harvested for each treatment group at each time point as described in Section 2.4. Messenger RNA was then reverse transcribed to cDNA and immune genes of interest were quantified by qPCR using the primers described in Table 4 and methods described in Section 2.4.

### 2.9 Use of IFNL3 for prevention of kennel cough virus infection of ALI-CRECs

To determine the value of canine IFNL3 for prevention infection with CAV-2, CDV, CaHV-1 or CIV, four groups (per virus) of differentiated ALI-CRECs from 3 dogs per treatment group were established: 1) Media only group (Controls), 2) IFNL3 treated group (IFNL3), 3) Virus infected group (CAV-2, CDV, CaHV-1, or CIV) and 4) IFNL3 treatment followed by virus infection with either CAV-2, CIV, CaHV-1 or CDV (CAV-2+IFNL3, CIV+IFNL3, CaHV-1+IFNL3 or CDV+IFNL3). For the experiments, plate wells and transwells were washed twice with 1X sterile PBS before interferon lambda 3 treatment or media respectively. For IFNL3 treatment, 100ug/ml of IFNL3 was added to the transwell for one hour before removal of the treatment inoculum and replacement with media and incubation for another 23 h before infection with the respective viruses (CAV-2, CDV, CaHV-1, CIV) at an MOI of 0.1 or mock infection as described in Section 2.5. Cells and supernatants were collected at 1 h post infection (hpi), 24 hpi, 48 hpi, 72 hpi and 96 hpi with the respective viruses. Collection of cells occurred by addition of accumax and centrifugation, followed by resuspension in 1 ml of 1X PBS. From 1 mL of resuspended cell pellet, 600uL were aliquoted for mRNA extraction to determine interferon and cytokine responses (described in Section 2.10), 200 μL were aliquoted for quantification of viral DNA/RNA, and 200 μL were aliquoted for virus titration using TCID50 assays. For quantification of viral DNA/RNA in cells or supernatants, viral RNA/DNA was isolated as described in Section 2.6 and virus specific qPCR assays were performed. Each reaction was carried out in 20 μl as follows: For quantification of CAV-2, SYBR Green E3 and U Exon gene-based primers were used as previously described (Balboni et al., 2015). For quantification of CaHV-1 DNA, Taqman based primers and probes targeting the gB gene of CaHV-1 and qPCR were used as previously described (Decaro et al., 2010). For quantification of CDV and CIV RNA qPCR assays using primers and probes targeting the N gene for CDV and M gene for CIV were used as previously published (Elia et al., 2006; Lin et al., 2012). For conversion of viral RNA into cDNA, AgPath-ID™ One-Step RT-PCR Reagents (Applied Biosystems, Foster City, CA) were used with the following cycling conditions: Initial thermal cycle: 45  °C for 10 min, 95  °C for 15 min followed by 45 cycles at 95  °C for 15 *sec*, 55  °C for 30 *sec*, 72  °C 10 *sec*. Sequence of primers and probes for viral qPCR assays are shown in Table 3. Intracellular and extracellular viral titers were determined as previously described (Tai et al., 2016). Briefly, virus titrations were performed in 96 well plates, and the TCID 50/mL-based titers were calculated using the Reed & Muench method as previously described (Reed and Muench, 1938).

**Table 3:** Primer and probe sequences used to amplify the viral DNA of CAV-2 and CaHV-1 and viral RNA of CDV, CIV using qPCR.

Viral qPCR	Forward Primer (5′-3′)	Reverse Primer (5′-3′) &	Probe	Reference
Canine distemper virus	AGCTAGTTTCATCTTAACTATCAAATT	TTAACTCTCCAGAAAACTCATGC	FAM-ACCCAAGAGCCGGATACATAGTTTCAATGC-TAMRA	Elia G et al., 2006
Canine herpesvirus 1	ACAGAGTTGATTGATAGAAGAGGTATG	CTGGTGTATTAAACTTTGAAGGCTTTA	6FAMTCTCTGGGGTCTTCATCCTTATCAAATGCGBHQ1	Decaro N et al., 2010
Canine adenovirus 2	CTGASACTGCWATRMCTATATAYATTTCCA	GACATAGARACRCAGGACCCAGA	-	Balboni A et al., 2015
Canine influenza virus (H3N2)	TCTATCGTCCCATCAGGC	GGTCTTGTCTTTAGCCATTC	-	Lin Y et al., 2012

### 2.10 Determination of interferon-stimulated gene and cytokine mRNA expression associated with infection and IFNL3 treatment

For determination of mRNA expression of interferon-stimulated genes (IFNL3, IFNK, IRF-7, MXI, OASI, VIPERIN) and other cytokines (IL-8, IL-10, TNF-alpha) following treatment with IFNL3 and/or infection with CAV-2, CDV, CaHV-1 or CIV, mRNA was isolated from the 600ul aliquot of cell suspensions harvested from each treatment group and time point as described in Section 2.4. Messenger RNA was reverse transcribed to cDNA and immune genes of interest were quantified by qPCR using the primers described in Table 4 and methods described in Section 2.4. To determine differences in mRNA expression, fold changes were calculated after normalizing cycle thresholds against the housekeeping gene GAPDH and by using the 2− ΔΔCt comparative Ct method (Schmittgen and Livak, 2008).

**Table 4:** Primer sequences used to quantify fold increase of interferon stimulated genes and cytokines in CAV-2, CDV and CaHV-1 virus ALI-CRECs (and CAV-2+IFNL3, CDV+IFNL3, CaHV-1+IFNL3 treated groups) by qPCR.

Gene	Forward Primer (5′-3′)	Reverse Primer (5′-3′)	Reference
IFN-lambda 3	TTGGGGTCCTCGCAGATGCAC	GCTGCTGCAGGTCCCAGGTC	Ichihashi T et al., 2013
ISIG 15	TCCTGGTGAGGAACCACAAGGG	TTCAGCCAGAACAGGTCGTC	Hao et al., 2022
IRF7	GCAAGGTCTACTGGGAGGTG	GTGCTGAAGTCGAAGATGGGG	Quinlan et al., 2020
IFNK	TGGAAGAGGATGGGATAAAC	TCTGATTTCCACTCGGATG	Luff et al., 2013

MXI	TTGAGGACCACCCACATTTC	CAGAGGCAGGGTTTTACAGATG	Ichihashi T et al., 2013
OASI	CTGTGCGGGTGTCTAAAGTTG	GAGCTGCTCCTGAAAACTCTTG	

VIPERIN	AGATTAAAGCCCTGAACCC	TCATCGCTGATAACAAACC	Zhang et al., 2017

IL-8	CAAGAGCCAGAAAGAAACCAGAAC	AAAGCTGCCAAGAGAGCAACA	Wang et al., 2007
IL-10	CGCTGTCACCGATTTCTTCC	CTGGAGCTTACTAAATGCGCTCT	
TNF-alpha	GAGCCGACGTGCCAATG	CAACCCATCTGACGGCACTA	

GAPDH	TCCTGCACCACCAACTGCTT	GTCTTCTGGGTGGCAGTGAT	Ichihashi T et al., 2013

### 2.11 Statistical analysis

Statistical analysis was performed using GraphPad PRISM version 9.5.1 (GraphPad Software, Inc., San Diego, CA) and a p-value < 0.05 was considered statistically significant. After determining that data was normally distributed using a Shapiro-Wilk test in prism to test all data for normality or lognormality, a two-way ANOVA with a multiple comparison Bonferroni correction was performed to identify differences in mRNA expression of immune markers in tracheal tissue, CRECs and ALI-CRECs. A paired T-test with a Benjamini, Krieger, and Yekutieli correction for multiple comparison was performed to compare interferon gene/cytokine/chemokine mRNA expression between ALI-CREC’s treated with or without canine IFNL3 at each time point. Similarly, a paired T-test with a Benjamini, Krieger, and Yekutieli correction for multiple comparison was performed to compare viral DNA/RNA detection by real-time PCR or viral titers between IFNL3 treated and untreated wells at each time point for the infection experiments with the respective viruses. A two-way ANOVA with Tukey multiple comparison tests were done to identify statistical differences in mRNA expression between media only, virus only, and IFNL3+ virus groups. In addition, multiple paired *t*-tests with Benjamini, Krieger, and Yekutieli correction for multiple comparison to adjust p-values were used to compare cytokine mRNA expression for each treatment group at each time point with pre-treatment values.

## 3 Results

### 3.1 ALI-CRECs resemble the natural airway morphologically

**Fig. 1:**
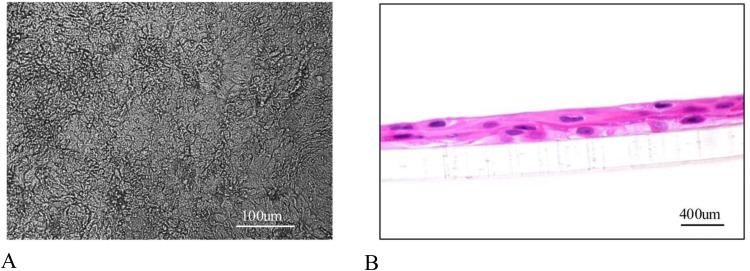
**Representative image of fully differentiated ALI-CRECs grown on a transwell insert two weeks post seeding. A.** Image taken with Leica inverted microscope at 100X magnification. **B.** Hematoxylin and eosin-stained image showing cross-sectioned transwell inserts of two-week-old, differentiated ALI-CRECs isolated from canine trachea. Image taken at 400X magnification.

Fig. 1 shows a microscopic and histologic representative image of canine respiratory epithelial cells collected and cultured from tracheas of dogs. After plating in transwells, around 80–90 % of ALI-CRECs attached within 24 h, and it took approximately 7 days to reach full confluency. Within 1–2 weeks, ALI-CRECs grew into a 3D multilayered epithelium that resembled the cell structure of the natural airway system morphologically (Fig. 1). Microscopically, the cells represented a pseudostratified epithelium that secreted mucous and contained goblet cells, similar to what can be seen in the natural airway of dogs as has been described previously (Shin et al., 2022; Runft et al., 2023). In contrast to the natural airway, presence of cilia was only observed occasionally at two weeks of culture, confirming previous reports by Runft et al. (Runft et al., 2023).

### 3.2 Canine respiratory epithelial cells retain expression of innate immune response-related genes observed in natural airway tissues

**Fig. 2:**
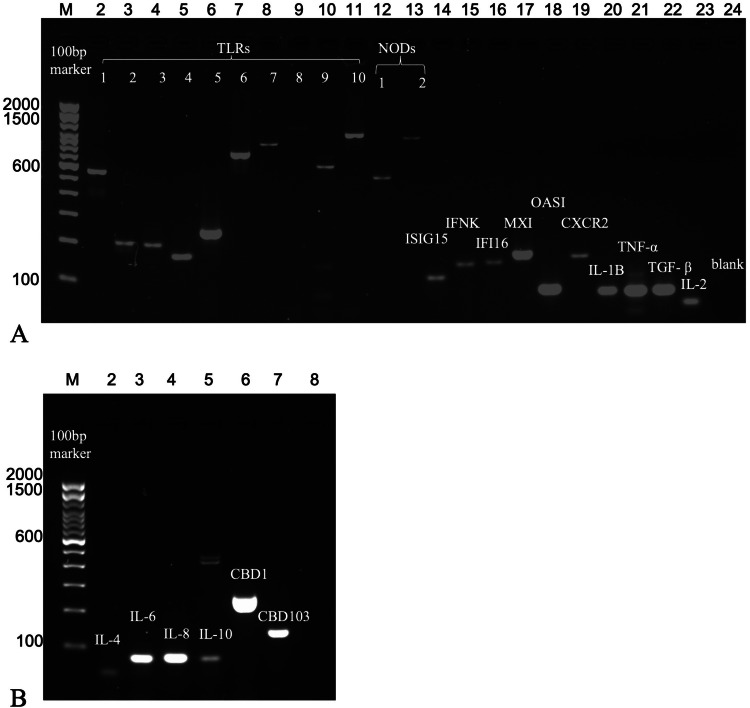
**Representative gel image showing amplified PCR Products of ALI-CRECs using 2 % agarose gel electrophoresis. A.** Lane M:100 bp ladder, lane 2–11: TLRs (TLR1-TLR10), lane 12 &13: NOD1 & NOD2, lane 14 &15: ISIG15 &IFNK, lane 16: IFI16, lane 17 & lane 18: MXI & OASI, lane 19: CXCR2, lanes 20, 21, 22 & 23: IL-1β, TNF-α, TGF- β & IL-2, lane 24: negative. **B**: Lane M: 100 bp ladder, lane 2–5: IL-4, IL-6, IL-8 & IL-10, lanes 6 & 7: CBD 1 & CBD 103, lane 8: negative.

To compare the expression of immune response-related genes in fresh or cultured canine respiratory epithelial cells with natural airway tissues, gene expression for 28 genes was analyzed using PCR in tracheal tissues (*n* = 8), freshly isolated CRECs (*n* = 5), and ALI-CRECs (*n* = 6) collected from dogs within one hour of euthanasia. Overall pattern recognition receptor (PRRs), interferon stimulated gene (ISG), chemokine, pro-inflammatory gene, β-defensin and other regulatory gene expression was detected in all groups (tracheal tissues, freshly isolated CRECs, and ALI-CRECs) but individual animal variability was high (Fig. 2, Table 5). Gene expression appeared to be most consistent in freshly isolated CRECs and ALI-CRECs, while expression in whole tracheal tissues was often only detected from less than half of the collected tissues for individual genes (Table 5).

**Table 5:** Immunological profiling of immune markers by PCR. The table represents number of positives for each gene in tracheal tissues, CRECs and ALI-CRECs per total number of dogs (n). A representative gel image is shown in Fig. 2.

Immune Markers	Name of Genes	Size of PCR Product (bp)	Samples		
			Dog Tracheas (*n* = 8)	Freshly Isolated CRECs (*n* = 5)	ALI-CRECs (*n* = 6)
Pattern Recognition Receptors	TLR 1	569	4/8	5/5	6/6
	TLR 2	211	4/8	5/5	6/6
	TLR 3	211	1/8	4/5	4/6
	TLR 4	174	5/8	4/5	4/6
	TLR 5	249	2/8	4/5	6/6
	TLR 6	770	5/8	5/5	5/6
	TLR 7	868	6/8	4/5	3/6
	TLR 8	1140	4/8	2/5	6/6
	TLR 9	612	5/8	4/5	6/6
	TLR 10	1047	4/8	4/5	5/6
	NOD 1	539	3/8	4/5	5/6
	NOD 2	968	4/8	3/5	3/6
Interferons & Interferon Induced Genes	IFN-K	149	7/8	4/5	5/6
	ISIG15	116	4/8	4/5	5/6
	IFI16	154	1/8	2/5	5/6
	MX1	181	1/8	2/5	6/6
	OAS1	87	1/8	4/5	6/6
Chemokine	CXCR2	165	5/8	4/5	6/6
Pro-inflammatory Genes	IL-1 β	80	6/8	2/5	6/6
	IL-2	80	1/8	2/5	6/6
	TNF-α	79	4/8	2/5	5/6
β Defensins	CBD1	256	1/8	4/5	6/6
	CBD103	143	1/8	3/5	6/6
Other Regulatory markers	IL-4	83	1/8	2/5	4/6
	IL-6	78	1/8	2/5	6/6
	IL-8	81	1/8	5/5	6/6
	IL-10	78	1/8	2/5	6/6
	TGF- β	79	1/8	2/5	6/6

**Fig. 3:**
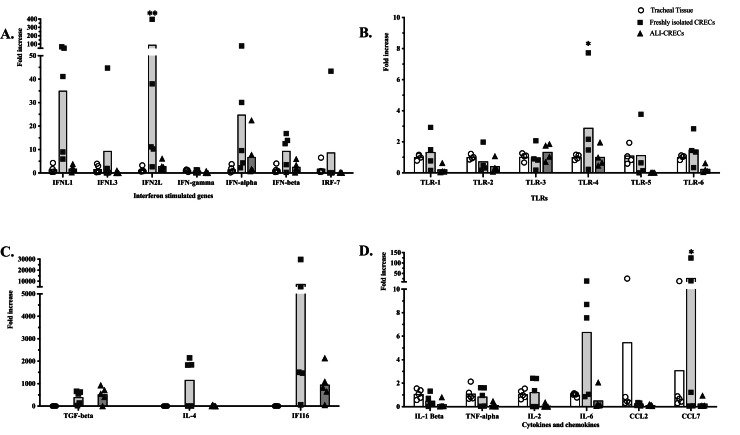
**Comparison of immune gene mRNA expression in tracheas, ALI-CRECs and freshly isolated CRECs. A.** Interferon stimulated genes. **B.** TLRs. **C.** TGF-beta, IL-4 & IF116. **D.** Cytokines and chemokines. Data is represented as average/tissue and symbols represent individual animals Circles represent tracheal tissue (*n* = 5), Squares represent freshly isolated respiratory epithelial cells (*n* = 5), Triangles represent ALI-CRECs (*n* = 5) of each animal. Fold changes compared to expression in tracheas were calculated using the 2-DDCt method after normalization with GAPDH. Significance was determined by two-way ANOVA with a multiple comparison Bonferroni correction ** *p* < 0.001, * *p* < 0.05.

To compare relative mRNA expression levels of individual genes in tracheal tissue, freshly isolated CRECs and ALI-CRECs, we analyzed the mRNA expression levels of interferon-stimulated genes, PRRs, cytokines, and chemokines by qPCR (Fig. 3). Overall, there were no significant differences observed in mRNA expression levels of the immune markers tested except for TLR4, IFN2L & CCL7 which were significantly higher in freshly isolated CRECs when compared to tracheal tissues or ALI-CRECs (p-value ≤ 0.05) (Fig. 3).

### 3.3 ALI-CRECs support infection with CAV-2, CDV, CaHV-1 and CIV

**Fig. 4:**
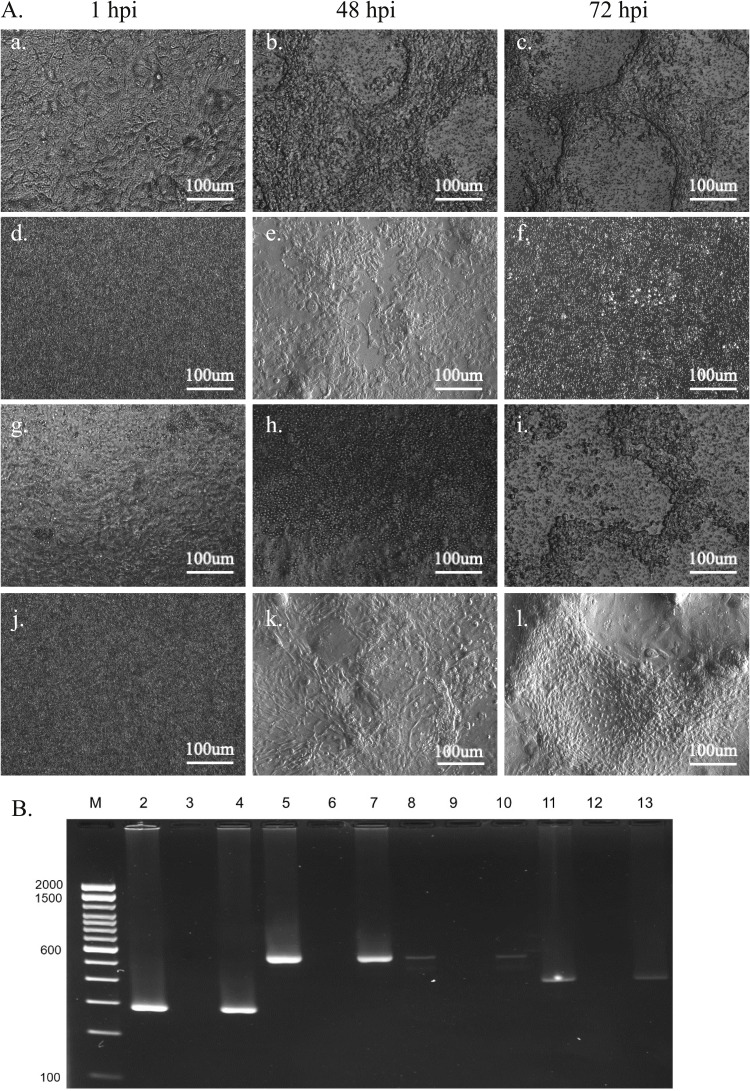
**Infection of ALI-CRECs with CRID viruses: A.** Representative image showing ALI-CRECs infected with CDV (a-c), CIV (d-f), CAV-2(g-i) and CaHV-1 (j-l) at 0, 48 and 72 hpi. All images were taken at 100 X magnification with a Leica inverted microscope. **B.** Representative gel image showing the presence of each virus’ genes measured by PCR: Lane M:100 bp marker (Trackit), lane 2: CDV positive control grown in Vero SLAM cells, lane 3: negative control, lane 4: ALI-CRECs positive for CDV (287 bp), lane 5: CIV positive control grown in MDCK, lane 6: negative control, lane 7: ALI-CRECs positive for CIV (544 bp), lane 8: CHV-1 positive control grown in MDCK, lane 9: negative control, lane 10: ALI-CRECs positive for CHV-1 (498 bp), lane 11: CAV positive control grown in MDCK, lane 12: negative control, lane 13: ALI-CRECs positive for CAV (398 bp).

To confirm that ALI-CRECs support infection with canine adenovirus (CAV-2), canine distemper virus (CDV), canine influenza virus (H3N2), and canine herpesvirus (CaHV-1), ALI-CRECs were infected with each virus at an MOI of 0.1 and changes were observed and recorded daily (Fig. 4A). All four viruses replicated efficiently, and cytopathic effects was observed microscopically in ALI-CRECs by 48 h post inoculation for each virus. For CDV and CaHV-1 syncytium formation was detected starting at 48 hpi (Fig. 4A, panel b for CDV, panel k for CaHV-1). For CIV, cell detachment was observed by 48 hpi (Fig. 4A, panel e) and for CAV-2, a rounding of infected cells was observed by 48 hpi followed by cell detachment observed by 72 hpi (Fig. 4A, panels h & g). These changes became become prominent by 72 hpi (Fig. 4A panels c, f, i, l). End point viral titers in ALI-CRECs infected with CAV-2 were 7 × 10^6 TCID_50_/ml, for CDV they were 1.0 × 10^6 TCID_50_/ml, for CaHV-1 they were 3.0 × 10^5 TCID_50_/ml, and for CIV they were 3.6 × 10^5TCID_50_/ml. Bands of the expected size were detected using agarose gel imaging followed by virus- specific by PCRs for each virus (Fig. 4B).

### 3.4 Only transient exposure of ALI-CRECs with IFNL3 was well tolerated

Preliminary experiments were conducted to determine a dose and treatment regimen with IFNL3 that would maintain viability of ALI-CRECs for 72 h (to allow for studying viral replication) and induce expression of interferon stimulated genes. We found that the continuous exposure of cells with recombinant protein led to changes in cell morphology consistent with cell death by 72 h post treatment (supplemental figure 1). While this likely does not reflect conditions in vivo, where natural cell turnover and proliferation ensure continued presence of an epithelial barrier and is part of the natural defense, this necessitated an alternative regimen for the in vitro experiments, where unchanged cell viability prior to infection is important. Because of this, we developed a regimen where ALI-CRECs were treated with different doses of IFNL3 for 1 hour (transient exposure). Using this regimen, we do not find any morphological changes or cell death at 24 and 48 h at any concentration, and only slight morphological changes started appearing at 72 h only in the 250ug/ml concentration (supplementary figure 2). Because, the focus of this study was to establish evidence of antiviral effects and induction of interferon responses prior to future follow-up study using whole animal models, we decided to use a concentration of 100ug/ml concentration for 1 hour followed by removal of IFNL3 and incubation in media or infection with the respective viruses for the remaining experiments in the study.

### 3.5 Treatment of ALI-CRECs with IFNL3 elevated interferon mRNA gene expression of interferon stimulated genes for up to 48 h

**Fig. 5:**
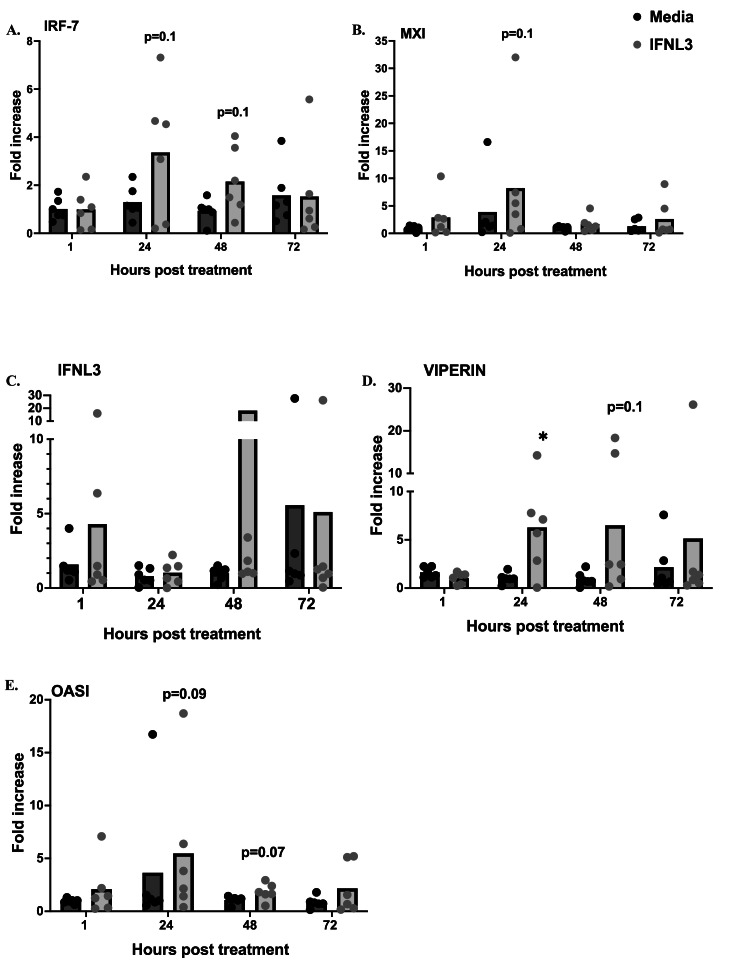
**Effect of IFNL3 treatment on mRNA expression of interferon stimulated genes in ALI-CRECs (*n*=6); a)** IRF-7, **b)** MXI, **c)** IFNL3, **d)** VIPERIN and **e)** OASI. A fold increase over untreated cells was calculated using the 2-DDCt method after normalizing the cycle thresholds (CT) with the housekeeping gene GAPDH. Data is represented as mean values with symbols representing individual animals. Significance was determined by paired T-test with a Benjamini, Krieger, and Yekutieli correction for multiple comparison to compare cytokine mRNA expression between IFNL3 treated and untreated cells at each time point, ** *p* < 0.001, * *p* < 0.05.

The effect IFNL3 treatment at a concentration of 100ug/ml on mRNA expression of interferon and other immune related genes in ALI-CRECs is shown in Fig. 5. No significant effect on mRNA expression of IFNK, ISIG15, TNF-alpha, IL-8 and IL-10 was observed at any time point (data not shown). When comparing IFNL3 treated ALI-CRECs with media controls we found that IFNL3 treatment increased IRF-7 mRNA expression at 24 and 48 h post treatment (hpt) (Fig. 5a) and MXI mRNA expression at 24 hpt (Fig. 5b) although increases did not reach statistically significance (*p* = 0.1). Viperin mRNA expression was significantly increased at 24 hpt (*p* = 0.04) and increases in mRNA expression were maintained at 48 and 72 hpt although not reaching statistical significance at these time points (Fig. 5d). Finally, increases in OAS mRNA expression were observed at 24 and 48 hpt (*p* = 0.09 and *p* = 0.07).

### 3.6 Treatment with IFNL3 significantly reduced CAV-2, CDV and CaHV-1 viral titers and CAV-2 DNA and CDV RNA

**Fig. 6:**
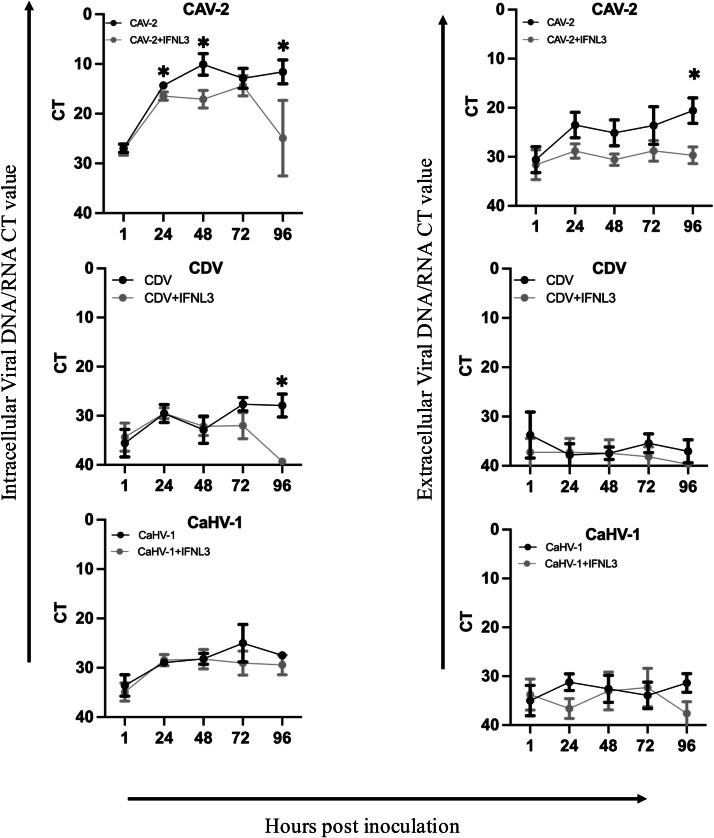
**Effect of IFNL3 treatment on viral nucleic acid of CAV-2, CaHV-1, and CDV (*n*=3)**. Intracellular viral nucleic acid levels, and extracellular viral nucleic acid levels are represented as mean values (± SEM) for each virus group and each virus+IFNL3 treated group. Significant differences of viral DNA/RNA in virus groups compared to the virus+IFNL3 group at each time point were determined by paired T-test with a Benjamini, Krieger, and Yekutieli correction for multiple comparison, ** *p* < 0.001, * *p* < 0.05.

**Fig. 7:**
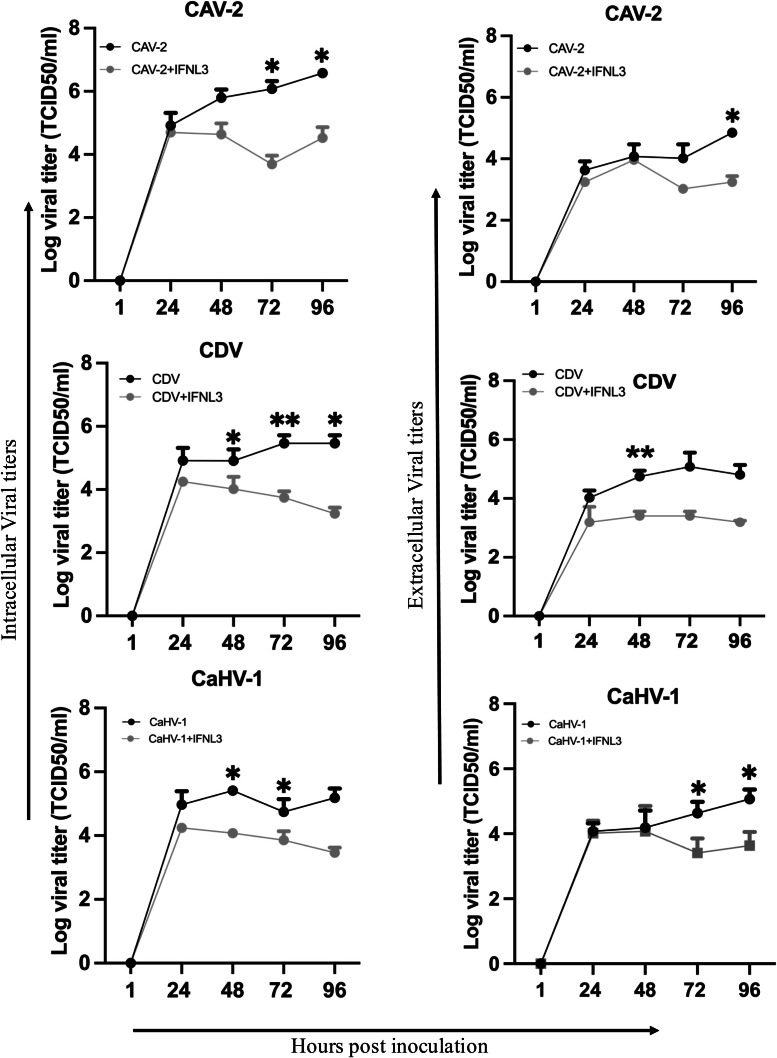
**Effect of IFNL3 treatment on viral titers for CAV-2, CaHV-1, and CDV (*n*=3)**. Log intracellular viral titers and log extracellular viral titers are represented as mean log values (± SEM) for log viral titers in the virus groups and virus+IFNL3 treated groups. Significant difference between virus only and virus+IFNL3 group at different time points: ** *p* < 0.001, * *p* < 0.05.

For determining the role of IFNL3 treatment in prevention/reduction of viral replication, viral nucleic acid, and infectious viral titers were quantified intracellularly (cells) and extracellularly (supernatants). Treatment of ALI-CRECs with IFNL3 significantly reduced viral DNA levels for CAV-2 by 24 hpi intracellularly, and 96 hpi extracellularly, and intracellular viral RNA levels for CDV by 96 hpi (Fig. 6). No significant differences were observed in viral DNA levels for CaHV-1 (Fig. 6) or in viral RNA levels for CIV (supplementary figure 3). Infectious viral titers were significantly reduced intra- and extracellularly for CAV-2, CDV, and CaHV-1 with the effects being more prominent and seen earlier intracellularly (Fig. 7). No effect of IFNL3 treatment was observed for CIV titers (supplementary figure 3). Interestingly, infection with CIV resulted in peak RNA and viral titers by 24 hpi intracellularly, while infection with the other viruses did not reach peak titers until later in the time course of infection.

### 3.7 Effect of interferon lambda 3 on immune gene mRNA expression during infection with CAV-2, CDV and CaHV-1

**Fig. 8:**
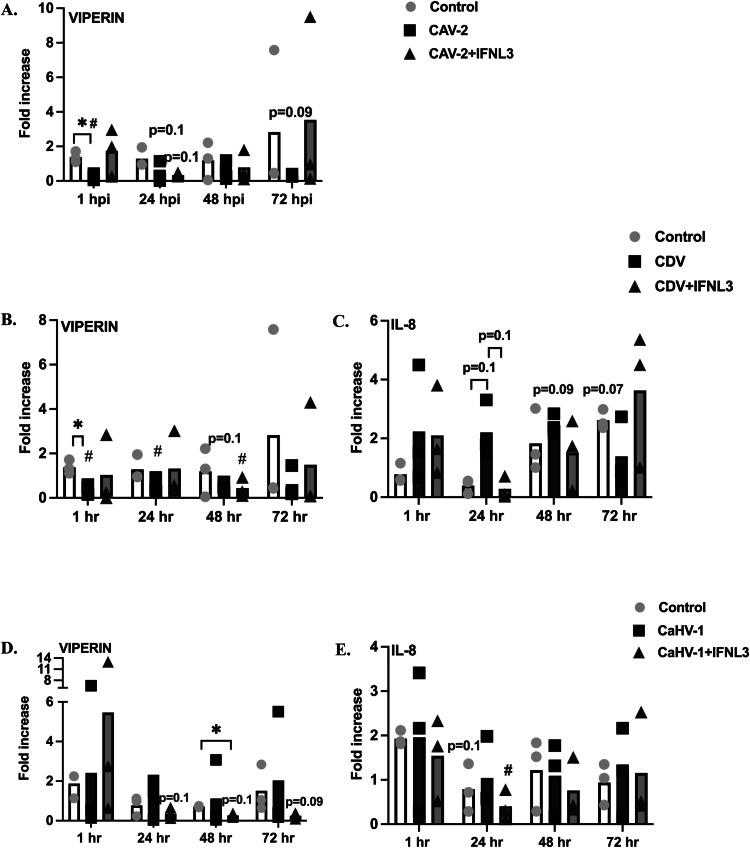
**Viperin and IL-8 mRNA expression virus infected ALI-CRECs compared to IFL3 treated and virus infected ALI-CRECs (*n*=3). A.** Viperin mRNA expression in controls, CAV-2 infected, and IFNL3 treated and CAV-2 infected ALI-CRECs. **B.** Viperin mRNA expression in controls, CDV infected, or in IFNL3 treated and CDV infected ALI-CRECs. **C.** IL-8 mRNA expression in controls, CDV infected, or IFNL3 treated and CDV infected ALI-CRECs. **D.** Viperin mRNA expression in controls, CaHV-1 infected, or in IFNL3 and CaHV-1infected ALI-CRECs. **E.** IL-8 mRNA expression in controls, CaHV-1 infected, or IFNL3 and CaHV-1 infected ALI-CRECs. Significant differences compared to pretreatment values were determined by multiple paired *t*-tests with Benjamini, Krieger, and Yekutieli correction and are indicated by #, *p* < 0.05. Significant differences between groups at the same time point are indicated by *, *p* < 0.05 and were determined by two-way ANOVA with Tukey multiple comparison tests.

To further characterize the effect of IFNL3 on the mRNA expression of cytokines (TNF-alpha, IL-8, IL-10) and interferon-stimulated genes (IFNL3, IFN-K, IRF-7, MX, OASI, viperin) during viral infection in ALI-CRECs, fold-changes in expression compared to controls were calculated for each virus infection alone or for IFNL3 treatment followed by infection for each gene. Overall, changes observed did often not reach statistical significance due to high variability between animals. Nevertheless, we found that there was a mild induction of IRF7, IFNL3 and IL-10 mRNA expression in CAV-2 and CAV-2 +IFNL3 infected cells and a slight reduction in IFNK mRNA expression (data not shown). Moreover, there was a reduction in viperin mRNA expression observed in CAV infected cells when compared to controls for 72 hpi. This reduction was significant at 1 hpi and approaching significance at 24 and 72 hpi (*p* ≤ 0.05, *p* = 0.1, *p* = 0.09) (Fig. 8a). Interestingly, this reduction was not observed for CAV-2 +IFNL3 treated cells.

Similarly, there was a mild induction of TNF-alpha for 48 hpi and a slight reduction of IFNK mRNA expression at all time points in the CDV and CDV+IFNL3 groups (data not shown). Similarly to what was observed with CAV-2, there was a significant reduction in viperin mRNA expression compared to levels in controls in both CDV and CDV+IFNL3 groups at different times (Fig. 8b). Moreover, IL-8 expression was induced in the CDV group at 24 and 48 hpi (*p* = 0.1, *p* = 0.09) when compared to controls while this effect was not observed in the CDV+IFNL3 group. Expression of other examined genes was unremarkable in CDV and CDV+IFNL3 groups (data not shown).

For CaHV-1, IFNK and MXI mRNA expression was significantly decreased in CaHV-1+IFNL3 treated cells compared to controls by 48 hpi (data not shown). This decrease was not observed in any other groups. Like CDV, viperin was significantly decreased in CaHV-1+IFNL3 group at 48 hpi (Fig. 8c). For IL-8, a significant reduction in mRNA expression of the CaHV-1+ IFNL3 group was observed at 48 hpi (Fig. 8c), but this reduction was not significant at any other time point (Fig. 8c). Similarly to the data seen with the other viruses, expression of other examined genes was unremarkable in CaHV-1 and CaHV-1+IFNL3 groups (data not shown).

## 4 Discussion

This study highlights the use of canine respiratory epithelial cell culture systems grown at the air liquid interface (ALI-CRECs) as an alternative ethical model for studying viruses associated with CIRDC and evaluation of antivirals like IFNL3. We show that ALI-CRECs retain many of the morphological and immunological features characteristic of the natural airway. However, while presence of cilia has been described for ALI respiratory cultures from other species (Nelli et al., 2016; Soboll Hussey et al., 2014), well-developed cilia formation was not observed in ALI-CRECs in our study. An explanation for this is likely that for the first ∼21 days post seeding cells still proliferate, and it has been shown that cilia are often reabsorbed into the cell before or during cell division (Runft et al., 2023; Silva et al., 2023). ALI-CRECs in our study were used for experiments ∼ 14 days post- seeding because at this time-point cells still divide actively and the mucociliary clearance (MCC) system is still being developed. This is often ideal for studies with respiratory viruses because the cells show good susceptibility to infection, specifically those that have evolved mechanism that target airway cilia and impair MCC mechanism (Kamiya et al., 2020). However, while the presence of cilia was not consistent in all cultures at this point, ALI-CRECs had developed into a pseudostratified respiratory epithelium, producing mucus and containing goblet cells, thus overall exhibiting the morphological structure of the respiratory epithelium as described for ALI cultures in other species (Nelli et al., 2016; Quintana et al., 2011; Runft et al., 2022). Moreover, while canine ALI-cultures have been characterized extensively morphologically previously (Runft et al., 2023; Shin et al., 2022; Silva et al., 2023), to our knowledge, our study is the first to also characterize the ALI-CREC system immunologically, and to use it to study infection with CIRDC viruses and evaluate the antiviral potential of IFNL3.

As previously shown for equine and feline ALI cultures, ALI-CRECs express early innate immune genes (PRRs, interferon stimulated genes, proinflammatory cytokines, chemokines). This study evaluated and compared the mRNA expression levels in tracheal tissues, freshly isolated CRECs and ALI-CRECs and showed that mRNA expression of immune genes of ALI-CRECs are comparable to those expressed by the tracheal tissue. However, the mRNA expression levels of several interferon genes and a few other immune genes were higher in freshly isolated CRECs when compared to tracheal tissues or fully differentiated ALI-CRECs. An explanation for this maybe that the process of cell isolation from tracheal tissue represents stress for the cells that may have led to the transient activation of genes responsible for cell survival, adaptation, and immediate cellular needs. This was also observed in other studies where environmental factors (i.e. physical, chemical and mechanical) showed a critical impact on the fate and the functions of primary cells (Greaney et al., 2020). In a study of human tracheal epithelial cells and breast tissue, it has also been shown that the process of using enzymatic dissociation resulted in physiological stress that induced the expression of early immune markers and upregulated stress mediated genes. This data supports the observed elevated levels of immune markers in freshly isolated epithelial cells following incubation in dissociation medium in our study. However, expression returned to baseline levels when the cells were cultured in stable ALI conditions and fully differentiated, confirming previous reports (Adam et al., 2017; O'Flanagan et al., 2019; van den Brink et al., 2017). This is also highlighted by the fact that there are no significant differences in the immune gene expressions of differentiated ALI-CREC’s and tracheal tissues, confirming that ALI-CRECs represent the natural airway immunologically, similar to what has been described in other species (Nelli et al., 2016; Park et al., 2010; Quintana et al., 2011).

In agreement with studies in other species, our study showed that ALI-CRECs support infection with several canine respiratory viruses (Bordes et al., 2024; Nelli et al., 2016; Soboll Hussey et al., 2014). All viruses studied (CAV-2; CDV; CaHV-1 and CIV) replicate efficiently in ALI-CRECs resulting in the expected morphological changes. These changes included cytopathic effect for all viruses by 48 hpi, syncytia formation for CDV and CaHV-1, rounding of cells and cell detachment for CIV and CAV-2 and fusion of infected cells for CDV, as described following infection of these viruses in their natural host (Shin et al., 2022; Wang et al., 2020; Yoon et al., 2010). Furthermore, growth kinetics for each virus were consistent with what has been described for the respective viruses in permissive cell lines, (Mochizuki, 2006) and endpoint titers for each virus confirmed efficient replication in ALI-CRECs. Interestingly, Shin et. al reported that CDV infected fully developed and well-differentiated airway epithelial cells only after EGTA treatment that disrupted adherent tight junctions, while direct infection of CDV from the apical side was unsuccessful (Shin et al., 2022). In contrast, we did not encounter any difficulties infecting ALI-CRECs apically with CDV. This difference could be explained because we used ALI-CRECs at two weeks post seeding, while Shin et. al used 28 days old cells. In a study published by Plotkowski et.al it was found that the degree and the differentiation state of the respiratory epithelial cells can affect infection with viral and bacterial pathogens (Plotkowski et al., 1999). Another study also found that the differentiation state is critical for infection and toxicological studies (Ghio et al., 2013). In bovine ALI cultures it has been demonstrated that the differentiation state of ALI cultures impacts the response during both infectious and toxicology studies (Cozens et al., 2018). Because, it has been shown that the differentiation state of ALI-cultures modulates the expression of transporters and carriers for drug transport, for studies in human nasal epithelial cells the suitable period is considered to be between 10–14 days post seeding (Cho et al., 2011; Silva et al., 2023). Further studies to evaluate how differentiation stages of canine respiratory epithelial ALI-cultures impact infection with different pathogens should be considered.

After confirming ALI-CRECs mimicked the natural airway morphologically and immunologically and supported infection with the respective kennel cough viruses, our next objective was to determine the value of IFNL3 treatment for protection from these viruses. Using the ALI-CREC system, we showed that prophylactic IFNL3 treatment significantly reduced viral DNA (CAV-2) and viral RNA (CDV) starting at 24 hpi for CAV-2 and by 96 h hpi for CDV. Looking at viral titers, we showed a significant reduction in infectious viral titers for CAV-2, CDV and CaHV-1 intracellularly and extracellularly starting at 24 hpi. The more significant reduction in infectious viral titers when compared to a reduction of viral nucleic acid highlights that while PCR-based assays are simple, cost effective, and quick for diagnostic purposes, the presence of viral nucleic acid does not always correlate with presence of infectious virus. In our experiments the time lag of significant reduction in viral nucleic acid detection compared to the significant reduction of infectious virus indicates that, while IFNL3 application reduced the amount of infectious virus and replication by 24 hpi, viral nucleic acid of “inactivated virus” was likely still detected at earlier time points and a reduction in nucleic acid detection was not observed until virus replication had ceased and virus was cleared. Our results further confirm that presence of viral genetic material in PCR doesn’t define the functional and infective capacity of the virus that can cause progression of disease (Herrero Hernando et al., 2020).

IFNL3 administration is thought to lead to activation of IFN signaling pathways, which are known to play a crucial role in immune defense against viruses. Liu et. al summarized that IFN-λ not only limits the spread of viruses by interference in the different stages of the virus life cycle (budding, attachment, replication and egress), but also directly and indirectly effects the immune cells during virus-induced inflammation (Liu et al., 2024). Other studies in different in-vitro models have demonstrated the role of type III interferon system in the defense from virus infection (Ank et al., 2006; Wang et al., 2009). Interestingly, in our study we did not demonstrate an effect of IFNL3 treatment on CIV virus replication or presence of CIV RNA, which is in contrast with a study conducted in mice, showing a significant reduction of influenza A virus infection in mice. Further, a study using IFNL3 treatment of an immortalized canine respiratory epithelial cell line with an adenovirus vector expressing IFNL3, also showed protection from CIV (Davidson et al., 2016; Kim et al., 2021). An explanation for these differences may be that, although the cell line used in this study were stable and form polarized monolayers, they lack the functional complexity and cellular diversity of the natural airway system (Chalak et al., 2024). Moreover, the authors in this study used Ad-caIFNλ3 for inducing the expression of IFNL3 and stimulation of interferon stimulated genes against influenza virus, whereas in our study we used a recombinant IFNL3 protein and treatment only occurred for 1 hour. These methods of IFNL3 administration showed kinetic differences in eliciting immune responses because an adenoviral vector-based system functions by constantly releasing IFNL3 protein to generate an antiviral response for a longer period of time as compared to our study where cells were exposed to the recombinant protein for a short duration of time (Awakoaiye et al., 2025). While use of recombinant IFNL3 protein was effective against CAV-2; CDV and CaHV-1 in the present study, it might not have generated a sufficient immune response against influenza virus. This may be explained because hemagglutinin (HA) and neuraminidase (NA) bind to receptors on both ciliated and non-ciliated cells and this interaction initiates induction of immune responses and infection that disrupts ciliary function and mucociliary clearance, and promotes apoptosis in ciliated cells (Horton et al., 2025; Wu et al., 2016). In our study, CRECs were still proliferating, and cilia were not fully developed which may have impacted infection with CIV. Compared to the other viruses used, CIV reached peak titers earlier and this more aggressive infection with CIV may have led to suppression and lack of strong interferon induction. Thus, future studies should consider using ALI-CRECs at full differentiation (and cilia development), inoculation of ALI-CRECs at multiple MOIs, and detailed mechanistic examinations of CIV pathogenesis and modulation of IFN pathways as well as further optimization of IFNL3 treatment. Moreover, similar to our findings, Iskandar et al. showed that infants and children were more susceptible to influenza infection and showed only weak induction of type I responses following IFNL3 administration (Iskander et al., 2007). In the present study, the canine epithelial respiratory cells that we used were isolated from young dogs. Whether age may have contributed to the lack of effectiveness for CIV prevention in our study is unclear, but factors including differences in IFNL3 administration (Ad-IFNL3 versus recombinant IFNL3 protein), viral strain used for infection, differentiation state of cells, or the timing of treatment may all explain why IFNL3 treatment was not effective for prevention of CIV infection of ALI-CRECs in our hands. Future detailed mechanistic and comparative studies should further elucidate the contribution of each of these factors.

While the data looking at the direct effect of IFNL3 treatment on virus replication were straightforward in our system, an evaluation of immune gene mRNA expression was overall more variable. Nevertheless, we did find that treatment of ALI-CRECs with 100ug/ml of IFNL3 induced mRNA expression of interferon stimulated genes MXI, IRF-7 and viperin and viperin mRNA expression lasted for up to 48 hrs. However, looking at IFNL3 mRNA expression directly, no significant inductions were observed at any timepoint. This lack of IFNL3 expression may be explained by the fact that both the type III interferon system and the type I interferon system signal through the JAK-STAT pathway, which initiates the transcription of interferon stimulated genes. Induction of type I interferon inhibits and cross-reacts with the type III interferon system (Singh and Dass, 2018), which may explain the observed lack of IFNL3 mRNA induction in our experiments.

In a next step we aimed to explore possible immune mechanisms that were associated with the protective effects of IFNL3 treatment for CAV-2, CDV and CaHV-1. Looking at mRNA expression of interferon- stimulated genes (IFNL3, IFNK, ISG15, IRF-7, OASI, viperin, and MXI) and cytokines (TNF-alpha, IL-8, and IL-10) in virus infected, IFNL3 treated + virus infected and media control incubated ALI-CRECs, we found there to be a high level of overall variability in our data and a lack of statistical significance for most of the data. Interesting observations included an overall effect on viperin expression for CAV-2, CDV and CaHV-1 ± IFNL3 treated cells. This could be explained because gene expression of viperin is regulated by both IFN-dependent and IFN-independent pathways and the activation of interferon-stimulated genes depends on several cellular transcriptional factors, including manipulation of host machinery via viral replication, or due to interferon mediated downregulation (Rivieccio et al., 2006). Secondly, infection with some viruses also activate the proteosome, which results in degradation of viperin. Combined, this data and our results suggest that activation or reduction of viperin expression can be facilitated by IFN mediated and viral mechanisms and further investigation is warranted to elucidate virus and IFNL3 mediated effects on viperin expression (Chan et al., 2008).

Finally, there was a reduction of IL-8 in the CDV inoculated cells when compared to both controls and CDV plus IFNL3 treated cells at 24 and 48 h. Similarly, the CaHV-1 plus IFNl3 treatment group showed a decrease in viperin and IL-8 compared to CaHV-1 infection or media alone at multiple time points. However, mRNA expression data was overall variable, which may partially be explained by a small number of experimental repeats for this set of experiments (*n* = 3). A further mechanistic investigation, including protein data, and analysis of downstream gene induction should be completed to confirm and further elucidate these pathways. However, our project’s main aim was to characterize the ALI-CREC system, show its value for studying canine respiratory viruses and evaluate the potential of IFNL3 as an antiviral agent. The initial exploration of immune gene/interferon mRNA expression allowed for an identification of important pathways that are targeted by the examined viruses and IFNL3 can and can be studied in further detail in future studies. Additionally, evaluating earlier time points post- treatment and infection is likely warranted, as onset of interferon responses has been shown to occur within hours of infection or treatment (Attreed et al., 2024; Dassanayake et al., 2024).

In summary, we show that respiratory epithelial cells grown at the ALI are excellent and ethical systems to study the pathogenesis of virus infection and evaluate antiviral agents. Using this system, we find that the prophylactic treatment of ALI-CRECs with IFNL3 induced interferon stimulated genes for up to 48 hpt and significantly reduced viral DNA/ RNA and viral titers of CAV-2, CDV and CaHV-1 but not CIV. However, limitations of the present study are the prophylactic only IFNL3 exposure. While our data support further investigation of IFNL3 as a potential antiviral strategy in canine respiratory infections, future studies should explore safety and efficacy of IFNL3 in vivo, evaluate IFNL3 treatment after infection has occurred and examine detailed downstream mechanistic functions of IFNL3 in the defense against these respiratory viruses.

## Funding

This work was supported by the Morris Animal Foundation Grant D21CA-074, Michigan State University, College of Veterinary Medicine and the Department of Pathobiology and Diagnostic Investigation.

Supplementary figure 1. Evaluation of morphological changes in ALI-CRECs following continuous treatment with different doses of IFNL3 over 72 h. Representative images of ALI-CRECs treated with 0, 10, 100 and 250ug/ml of IFNL3 per transwell and incubated for 1, 24, 48, and 72 h.

Supplementary figure 2. Morphological evaluation of ALI-CRECs after a 1-hour transient incubation with different doses of IFNL3 over 72 h. ALI-CRECs were treated with 0, 10, 100 and 250ug/ml in each transwell and incubated for 1 hr before removal of IFNL3. Cells were subsequently cultured for another 1, 24, 48, and 72 h to determine morphological changes. Red arrows indicate morphological changes in the cells at a concentration of 250ug/ml cIFNL3 per transwell at 72hpi that were considered a sign of low toxicity.

Supplementary figure 3. Effect of IFNL3 treatment on viral nucleic acid and titers of CIV (*n* = 3). Intracellular and extracellular viral RNA levels and log CIV titers are represented as mean values (± SEM) for CIV infected ALI-CRECs and CIV+IFNL3 treated ALI-CRECs. Significant differences of viral RNA or viral titers in the CIV group compared to the CIV+IFNL3 group at each time point were determined by paired T-test with a Benjamini, Krieger, and Yekutieli correction for multiple comparison.

## CRediT authorship contribution statement

**Swati Sharma:** Writing – review & editing, Writing – original draft, Visualization, Validation, Methodology, Investigation, Formal analysis, Data curation, Conceptualization. **Glorián Berríos-Vázquez:** Writing – review & editing, Methodology, Investigation. **Kennedy Baldwin:** Methodology, Investigation, Data curation. **Roger Maes:** Writing – review & editing, Methodology, Funding acquisition, Data curation, Conceptualization. **Gisela Soboll Hussey:** Writing – review & editing, Visualization, Validation, Supervision, Resources, Project administration, Methodology, Investigation, Funding acquisition, Formal analysis, Data curation, Conceptualization.

## Declaration of competing interest

The authors declare the following financial interests/personal relationships which may be considered as potential competing interests:

Gisela Soboll Hussey reports financial support was provided by Morris Animal Foundation. If there are other authors, they declare that they have no known competing financial interests or personal relationships that could have appeared to influence the work reported in this paper.

## Data Availability

Data will be made available on request.

## Appendix Supplementary materials

Image, application 1

## References

[bib0001] Adam M., Potter A.S., Potter S.S. (2017). Psychrophilic proteases dramatically reduce single-cell RNA-seq artifacts: a molecular atlas of kidney development. Development.

[bib0003] Anderton T.L., Maskell D.J., Preston A. (2004). Ciliostasis is a key early event during colonization of canine tracheal tissue by Bordetella bronchiseptica. Microbiology.

[bib0004] Ank N., West H., Bartholdy C., Eriksson K., Thomsen A.R., Paludan S.R. (2006). Lambda interferon (IFN-lambda), a type III IFN, is induced by viruses and IFNs and displays potent antiviral activity against select virus infections in vivo. J. Virol..

[bib0005] Attreed S.E., Silva C., Rodriguez-Calzada M., Mogulothu A., Abbott S., Azzinaro P., Canning P., Skidmore L., Nelson J., Knudsen N., Medina G.N., de Los Santos T., Diaz-San Segundo F. (2024). Prophylactic treatment with PEGylated bovine IFNlambda3 effectively bridges the gap in vaccine-induced immunity against FMD in cattle. Front. Microbiol..

[bib0006] Avadhanula V., Rodriguez C.A., Devincenzo J.P., Wang Y., Webby R.J., Ulett G.C., Adderson E.E. (2006). Respiratory viruses augment the adhesion of bacterial pathogens to respiratory epithelium in a viral species- and cell type-dependent manner. J. Virol..

[bib0007] Awakoaiye B., Li S., Sanchez S., Dangi T., Irani N., Arroyo L., Arellano G., Mohammadabadi S., Aid M., Penaloza-MacMaster P. (2025). Comparative analysis of adenovirus, mRNA, and protein vaccines reveals context-dependent immunogenicity and efficacy. JCI. Insight..

[bib0008] Balboni A., Dondi F., Prosperi S., Battilani M. (2015). Development of a SYBR Green real-time PCR assay with melting curve analysis for simultaneous detection and differentiation of canine adenovirus type 1 and type 2. J. Virol. Methods.

[bib0009] Bordes L., Gerhards N.M., Peters S., van Oort S., Roose M., Dresken R., Venema S., Vrieling M., Engelsma M., van der Poel W.H.M., de Swart R.L. (2024). H5N1 clade 2.3.4.4b avian influenza viruses replicate in differentiated bovine airway epithelial cells cultured at air-liquid interface. J. Gen. Virol..

[bib0010] Buonavoglia C., Martella V. (2007). Canine respiratory viruses. Vet. Res..

[bib0011] Chalak M., Hesaraki M., Mirbahari S.N., Yeganeh M., Abdi S., Rajabi S., Hemmatzadeh F. (2024). Cell immortality: in vitro effective techniques to achieve and investigate its applications and challenges. Life.

[bib0012] Chan Y.L., Chang T.H., Liao C.L., Lin Y.L. (2008). The cellular antiviral protein viperin is attenuated by proteasome-mediated protein degradation in Japanese encephalitis virus-infected cells. J. Virol..

[bib1002] Chiang H.C., Wang Y.S., Chou C.H., Liao A.T., Chu R.M., Lin C.S. (2012). Overexpression of chemokine ligand 7 is associated with the progression of canine transmissible venereal tumor. BMC Vet. Res..

[bib0013] Cho H.J., Choi M.K., Lin H., Kim J.S., Chung S.J., Shim C.K., Kim D.D. (2011). Expression and functional activity of P-glycoprotein in passaged primary human nasal epithelial cell monolayers cultured by the air-liquid interface method for nasal drug transport study. J. Pharm. Pharmacol..

[bib0014] Clark A.B., Randell S.H., Nettesheim P., Gray T.E., Bagnell B., Ostrowski L.E. (1995). Regulation of ciliated cell differentiation in cultures of rat tracheal epithelial cells. Am. J. Respir. Cell Mol. Biol..

[bib0015] Cozens D., Sutherland E., Marchesi F., Taylor G., Berry C.C., Davies R.L. (2018). Temporal differentiation of bovine airway epithelial cells grown at an air-liquid interface. Sci. Rep..

[bib0016] Dassanayake R.P., Menghwar H., Bickel K.A., Holthausen D.J., Ma H., Diaz-San Segunda F., Rodriguez-Calzada M., Medina G.N., Attreed S., Falkenberg S.M., Kanipe C., Sacco R.E., De Los Santos T., Casas E. (2024). Antiviral activity of bovine type III interferon against bovine viral diarrhea virus is greatly reduced in bovine turbinate cells due to limited expression of IFN lambda receptor 1 (IL-28Ralpha). Front. Immunol..

[bib0017] Davidson S., McCabe T.M., Crotta S., Gad H.H., Hessel E.M., Beinke S., Hartmann R., Wack A. (2016). IFNlambda is a potent anti-influenza therapeutic without the inflammatory side effects of IFNalpha treatment. EMBo Mol. Med..

[bib0018] Day M.J., Carey S., Clercx C., Kohn B., MarsilIo F., Thiry E., Freyburger L., Schulz B., Walker D.J. (2020). Aetiology of canine infectious Respiratory disease complex and prevalence of its pathogens in Europe. J. Comp. Pathol..

[bib0019] Decaro N., Amorisco F., Desario C., Lorusso E., Camero M., Bellacicco A.L., Sciarretta R., Lucente M.S., Martella V., Buonavoglia C. (2010). Development and validation of a real-time PCR assay for specific and sensitive detection of canid herpesvirus 1. J. Virol. Methods.

[bib1006] Dong J., Tsui W.N.T., Leng X., Fu J., Lohman M., Anderson J., Hamill V., Lu N., Porter E.P., Gray M., Sebhatu T., Brown S., Pogranichniy R., Wang H., Noll L., Bai J. (2022). Development of a three-panel multiplex real-time PCR assay for simultaneous detection of nine canine respiratory pathogens. J. Microbiol. Methods.

[bib0020] Edinboro C.H., Ward M.P., Glickman L.T. (2004). A placebo-controlled trial of two intranasal vaccines to prevent tracheobronchitis (kennel cough) in dogs entering a humane shelter. Prev. Vet. Med..

[bib0022] Elia G., Decaro N., Martella V., Cirone F., Lucente M.S., Lorusso E., Di Trani L., Buonavoglia C. (2006). Detection of canine distemper virus in dogs by real-time RT-PCR. J. Virol. Methods.

[bib0021] Elia G., Camero M., Losurdo M., Lucente M.S., Larocca V., Martella V., Decaro N., Buonavoglia C. (2015). Virological and serological findings in dogs with naturally occurring distemper. J. Virol. Methods.

[bib1003] Erles K., Brownlie J. (2010). Expression of beta-defensins in the canine respiratory tract and antimicrobial activity against Bordetella bronchiseptica. Vet. Immunol. Immunopathol..

[bib0023] Farber I., Kruger J., Rocha C., Armando F., von Kockritz-Blickwede M., Pohlmann S., Braun A., Baumgartner W., Runft S., Kruger N. (2022). Investigations on SARS-CoV-2 susceptibility of domestic and wild animals using primary cell culture models derived from the upper and lower Respiratory tract. Viruses.

[bib0025] Ford R.B., Vaden S.L., Greene C.E. (1998). Infectious Diseases of the Dog and Cat.

[bib1004] Frisk A.L., König M., Moritz A., Baumgärtner W. (1999). Detection of canine distemper virus nucleoprotein RNA by reverse transcription-PCR using serum, whole blood, and cerebrospinal fluid from dogs with distemper. J. Clin. Microbiol..

[bib0026] Ghio A.J., Dailey L.A., Soukup J.M., Stonehuerner J., Richards J.H., Devlin R.B. (2013). Growth of human bronchial epithelial cells at an air-liquid interface alters the response to particle exposure. Part Fibre Toxicol..

[bib0027] Greaney A.M., Adams T.S., Brickman Raredon M.S., Gubbins E., Schupp J.C., Engler A.J., Ghaedi M., Yuan Y., Kaminski N., Niklason L.E. (2020). Platform effects on regeneration by pulmonary basal cells as evaluated by single-cell RNA sequencing. Cell Rep..

[bib0028] Gultom M., Licheri M., Laloli L., Wider M., Strassle M., V'Kovski P., Steiner S., Kratzel A., Thao T.T.N., Probst L., Stalder H., Portmann J., Holwerda M., Ebert N., Stokar-Regenscheit N., Gurtner C., Zanolari P., Posthaus H., Schuller S., Vicente-Santos A., Moreira-Soto A., Corrales-Aguilar E., Ruggli N., Tekes G., von Messling V., Sawatsky B., Thiel V., Dijkman R. (2021). Susceptibility of well-differentiated airway epithelial cell cultures from domestic and wild animals to severe acute Respiratory syndrome coronavirus 2. Emerg. Infect. Dis..

[bib75] Hao X., Li Y., Chen H., Chen B., Liu R., Wu Y., Xiao X., Zhou P., Li S. (2022). Canine circovirus suppresses the type I interferon response and protein expression but promotes CPV-2 replication. Int. J. Mol. Sci..

[bib0029] Herrero Hernando C., Alvarez Serra J.A., Elizari Saco M.J., Martinez-Nadal S., Vila Ceren C. (2020). PCR test for SARS-CoV-2 persistently positive. Virus detection is not always COVID-19. An. Pediatr. Engl. Ed..

[bib0030] Holmgren J., Czerkinsky C. (2005). Mucosal immunity and vaccines. Nat. Med..

[bib0031] Horton K., Wing P.A.C., Jackson C.L., McCormick C.J., Carroll M.P., Lucas J.S. (2025). Interplay between respiratory viruses and cilia in the airways. Eur. Respir. Rev..

[bib0032] Ichihashi T., Asano A., Usui T., Takeuchi T., Watanabe Y., Yamano Y. (2013). Antiviral and antiproliferative effects of canine interferon-lambda1. Vet. Immunol. Immunopathol..

[bib0033] Iskander M., Booy R., Lambert S. (2007). The burden of influenza in children. Curr. Opin. Infect. Dis..

[bib0034] Kamiya Y., Fujisawa T., Katsumata M., Yasui H., Suzuki Y., Karayama M., Hozumi H., Furuhashi K., Enomoto N., Nakamura Y., Inui N., Setou M., Ito M., Suzuki T., Ikegami K., Suda T. (2020). Influenza A virus enhances ciliary activity and mucociliary clearance via TLR3 in airway epithelium. Respir. Res..

[bib0036] Kim S.H., Jang Y.S. (2014). Antigen targeting to M cells for enhancing the efficacy of mucosal vaccines. Exp. Mol. Med..

[bib0035] Kim D.H., Park B.J., Ahn H.S., Go H.J., Kim D.Y., Kim J.H., Lee J.B., Park S.Y., Song C.S., Lee S.W., Choi I.S. (2021). Canine interferon lambda 3 expressed using an adenoviral vector effectively induces antiviral activity against canine influenza virus. Virus. Res..

[bib0037] Kohara J., Nishikura Y., Konnai S., Tajima M., Onuma M. (2012). Effects of interferon-tau on cattle persistently infected with bovine viral diarrhea virus. Jpn. J. Vet. Res..

[bib0038] Kumar S., Driskell E.A., Cooley A.J., Jia K., Blackmon S., Wan X.F., Uhl E.W., Saliki J.T., Sanchez S., Krimer P.M., Hogan R.J. (2015). Fatal canid herpesvirus 1 Respiratory infections in 4 clinically healthy adult dogs. Vet. Pathol..

[bib0039] Lazear H.M., Nice T.J., Diamond M.S. (2015). Interferon-lambda: immune functions at barrier surfaces and beyond. Immunity.

[bib0040] Lee Y., Maes R., Tai S.S., Soboll Hussey G. (2019). Viral replication and innate immunity of feline herpesvirus-1 virulence-associated genes in feline respiratory epithelial cells. Virus. Res..

[bib0041] Lin Y., Zhao Y., Zeng X., Lu C., Liu Y. (2012). Genetic and pathobiologic characterization of H3N2 canine influenza viruses isolated in the Jiangsu Province of China in 2009-2010. Vet. Microbiol..

[bib0042] Liu Y.G., Jin S.W., Zhang S.S., Xia T.J., Liao Y.H., Pan R.L., Yan M.Z., Chang Q. (2024). Interferon lambda in respiratory viral infection: immunomodulatory functions and antiviral effects in epithelium. Front. Immunol..

[bib77] Luff J.A., Yuan H., Suter M.M., Müller E.J., Schlegel R., Moore P.F. (2013). Canine keratinocytes upregulate type I interferons and proinflammatory cytokines in response to poly(dA:dT) but not to canine papillomavirus. Vet. Immunol. Immunopathol..

[bib0044] Marshall J.S., Warrington R., Watson W., Kim H.L. (2018). An introduction to immunology and immunopathology. Allergy Asthma Clin. Immunol..

[bib1000] Mercier E., Peters I.R., Day M.J., Clercx C., Peeters D. (2012). Toll- and NOD-like receptor mRNA expression in canine sino-nasal aspergillosis and idiopathic lymphoplasmacytic rhinitis. Vet. Immunol. Immunopathol..

[bib0045] Miller D.M., Klucher K.M., Freeman J.A., Hausman D.F., Fontana D., Williams D.E. (2009). Interferon lambda as a potential new therapeutic for hepatitis C. Ann. N. Y. Acad. Sci..

[bib0046] Mitchell J.A., Brownlie J. (2015). The challenges in developing effective canine infectious respiratory disease vaccines. J. Pharm. Pharmacol..

[bib0047] Mochizuki M. (2006). Growth characteristics of canine pathogenic viruses in MDCK cells cultured in RPMI 1640 medium without animal protein. Vaccine.

[bib0048] Nelli R.K., Maes R., Kiupel M., Hussey G.S. (2016). Use of a feline respiratory epithelial cell culture system grown at the air-liquid interface to characterize the innate immune response following feline herpesvirus 1 infection. Virus. Res..

[bib0049] O'Flanagan C.H., Campbell K.R., Zhang A.W., Kabeer F., Lim J.L.P., Biele J., Eirew P., Lai D., McPherson A., Kong E., Bates C., Borkowski K., Wiens M., Hewitson B., Hopkins J., Pham J., Ceglia N., Moore R., Mungall A.J., McAlpine J.N., Team C.I.G.C., Shah S.P., Aparicio S. (2019). Dissociation of solid tumor tissues with cold active protease for single-cell RNA-seq minimizes conserved collagenase-associated stress responses. Genome Biol..

[bib0050] Park K.U., Jin P., Sabatino M., Feng J., Civini S., Khuu H., Berg M., Childs R., Stroncek D. (2010). Gene expression analysis of ex vivo expanded and freshly isolated NK cells from cancer patients. J. ImmunOther.

[bib0051] Perez-Martin E., Weiss M., Diaz-San Segundo F., Pacheco J.M., Arzt J., Grubman M.J., de los Santos T. (2012). Bovine type III interferon significantly delays and reduces the severity of foot-and-mouth disease in cattle. J. Virol..

[bib0052] Plotkowski M.C., de Bentzmann S., Pereira S.H., Zahm J.M., Bajolet-Laudinat O., Roger P., Puchelle E. (1999). Pseudomonas aeruginosa internalization by human epithelial respiratory cells depends on cell differentiation, polarity, and junctional complex integrity. Am. J. Respir. Cell Mol. Biol..

[bib0053] Priestnall S.L., Mitchell J.A., Brooks H.W., Brownlie J., Erles K. (2009). Quantification of mRNA encoding cytokines and chemokines and assessment of ciliary function in canine tracheal epithelium during infection with canine respiratory coronavirus (CRCoV). Vet. Immunol. Immunopathol..

[bib0054] Prytherch Z., Job C., Marshall H., Oreffo V., Foster M., BeruBe K. (2011). Tissue-specific stem cell differentiation in an in vitro airway model. Macromol. Biosci..

[bib76] Quinlan S., May S., Weeks R., Yuan H., Luff J.A. (2020). Abrogation of constitutive and induced type I and type III interferons and interferon-stimulated genes in keratinocytes by canine papillomavirus 2 E6 and E7. Viruses.

[bib0055] Quintana A.M., Landolt G.A., Annis K.M., Hussey G.S. (2011). Immunological characterization of the equine airway epithelium and of a primary equine airway epithelial cell culture model. Vet. Immunol. Immunopathol..

[bib0056] Reagan K.L., Sykes J.E. (2020). Canine infectious Respiratory disease. Vet. Clin. North Am. Small. Anim. Pract..

[bib0057] Reed L.J., Muench H. (1938). A simple method of estimating fifty per cent endpoints. Am. J. Hyg..

[bib0058] Rivieccio M.A., Suh H.S., Zhao Y., Zhao M.L., Chin K.C., Lee S.C., Brosnan C.F. (2006). TLR3 ligation activates an antiviral response in human fetal astrocytes: a role for viperin/cig5. J. Immunol..

[bib0059] Rowe T., Fletcher A., Svoboda P., Pohl J., Hatta Y., Jasso G., Wentworth D.E., Ross T.M. (2024). Interferon as an immunoadjuvant to enhance antibodies following influenza B infection and vaccination in ferrets. NPJ Vaccines.

[bib0060] Runft S., Farber I., Kruger J., Kruger N., Armando F., Rocha C., Pohlmann S., Burigk L., Leitzen E., Ciurkiewicz M., Braun A., Schneider D., Baumgartner L., Freisleben B., Baumgartner W. (2022). Alternatives to animal models and their application in the discovery of species susceptibility to SARS-CoV-2 and other respiratory infectious pathogens: a review. Vet. Pathol..

[bib0061] Runft S., Farber I., Kruger J., Schone K., Lehmbecker A., Baumgartner W. (2023). In vitro characteristics of canine primary tracheal epithelial cells maintained at an air-liquid interface compared to In vivo morphology. Int. J. Mol. Sci..

[bib0062] Schmittgen T.D., Livak K.J. (2008). Analyzing real-time PCR data by the comparative C(T) method. Nat. Protoc..

[bib1005] Schulze C., Baumgärtner W. (1998). Nested polymerase chain reaction and in situ hybridization for diagnosis of canine herpesvirus infection in puppies. Vet. Pathol..

[bib0063] Shin D.L., Chludzinski E., Wu N.H., Peng J.Y., Ciurkiewicz M., Sawatsky B., Pfaller C.K., Baechlein C., von Messling V., Haas L., Beineke A., Herrler G. (2022). Overcoming the barrier of the Respiratory epithelium during Canine Distemper virus infection. mBio.

[bib1001] Silva E., Henriques S., Brito S., Ferreira-Dias G., Lopes-da-Costa L., Mateus L. (2012). Oestrous cycle-related changes in production of Toll-like receptors and prostaglandins in the canine endometrium. J. Reprod. Immunol..

[bib0064] Silva S., Bicker J., Falcao A., Fortuna A. (2023). Air-liquid interface (ALI) impact on different respiratory cell cultures. Eur. J. Pharm. Biopharm..

[bib0065] Singh P., Dass J.F.P. (2018). Nearly neutral evolution in IFNL3 gene retains the immune function to detect and clear the viral infection in HCV. Prog. Biophys. Mol. Biol..

[bib0066] Soboll Hussey G., Ashton L.V., Quintana A.M., Lunn D.P., Goehring L.S., Annis K., Landolt G. (2014). Innate immune responses of airway epithelial cells to infection with equine herpesvirus-1. Vet. Microbiol..

[bib0067] Tai S.H., Holz C., Engstrom M.D., Cheng H.H., Maes R.K. (2016). In vitro characterization of felid herpesvirus 1 (FHV-1) mutants generated by recombineering in a recombinant BAC vector. Virus. Res..

[bib0068] van den Brink S.C., Sage F., Vertesy A., Spanjaard B., Peterson-Maduro J., Baron C.S., Robin C., van Oudenaarden A. (2017). Single-cell sequencing reveals dissociation-induced gene expression in tissue subpopulations. Nat. Methods.

[bib79] Wang Y.S., Chi K.H., Chu R.M. (2007). Cytokine profiles of canine monocyte-derived dendritic cells as a function of lipopolysaccharide- or tumor necrosis factor-alpha-induced maturation. Vet. Immunol. Immunopathol..

[bib0069] Wang J., Oberley-Deegan R., Wang S., Nikrad M., Funk C.J., Hartshorn K.L., Mason R.J. (2009). Differentiated human alveolar type II cells secrete antiviral IL-29 (IFN-lambda 1) in response to influenza A infection. J. Immunol..

[bib1070] Wang C., Wang Q., Hu J., Sun H., Pu J., Liu J., Sun Y. (2017). A Multiplex RT-PCR Assay for Detection and Differentiation of Avian-Origin Canine H3N2, Equine-Origin H3N8, Human-Origin H3N2, and H1N1/2009 Canine Influenza Viruses. PLoS One.

[bib0070] Wang T.E., Chao T.L., Tsai H.T., Lin P.H., Tsai Y.L., Chang S.Y. (2020). Differentiation of cytopathic effects (CPE) induced by influenza virus infection using deep convolutional Neural networks (CNN). PLoS Comput. Biol..

[bib0071] Wu N.H., Yang W., Beineke A., Dijkman R., Matrosovich M., Baumgartner W., Thiel V., Valentin-Weigand P., Meng F., Herrler G. (2016). The differentiated airway epithelium infected by influenza viruses maintains the barrier function despite a dramatic loss of ciliated cells. Sci. Rep..

[bib0072] Ye L., Schnepf D., Staeheli P. (2019). Interferon-lambda orchestrates innate and adaptive mucosal immune responses. Nat. Rev. Immunol..

[bib0073] Yoon S.S., Byun J.W., Park Y.I., Kim M.J., Bae Y.C., Song J.Y. (2010). Comparison of the diagnostic methods on the canine adenovirus type 2 infection. Basic Appl Pathol.

[bib0074] Zarski L.M., Vaala W.E., Barnett D.C., Bain F.T., Soboll Hussey G. (2021). A live-attenuated equine influenza vaccine stimulates innate immunity in equine Respiratory epithelial cell cultures that could provide protection from equine herpesvirus 1. Front. Vet. Sci..

[bib78] Zhang Y., Zhu M., Li G., Liu J., Zhai X., Wang R., Zhang J., Xing G., Gu J., Yan L., Lei J., Sun H., Shi Z., Liu F., Hu B., Su S., Zhou J. (2017). .Identification and function analysis of canine stimulator of interferon gene (STING). Microb. Pathog..

