# Supplementary material for: IFNL3 reduces replication of CIRDC-associated viruses in canine airway epithelial cells
Source: Virus Res. 2026 May 3;369:199742. doi: 10.1016/j.virusres.2026.199742 (PMC13213814; doi:10.1016/j.virusres.2026.199742)
Supplement: Supplementary file 1 [file mmc1.pdf]

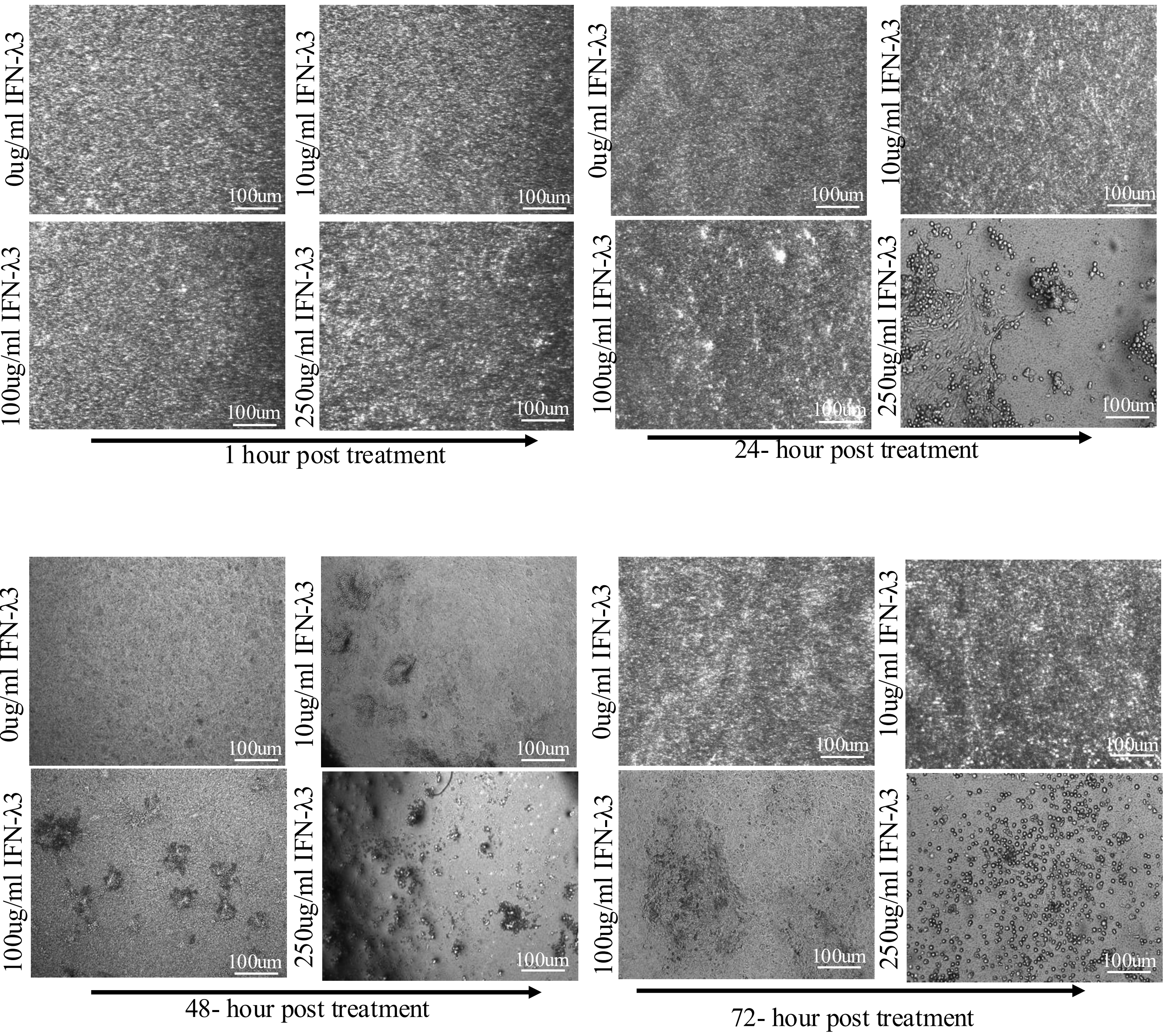
0ug/ml IFN-λ3
10ug/ml IFN-λ3
100ug/ml IFN-λ3
250ug/ml IFN-λ3
100um
1 hour post treatment
24- hour post treatment
48- hour post treatment
72- hour post treatment

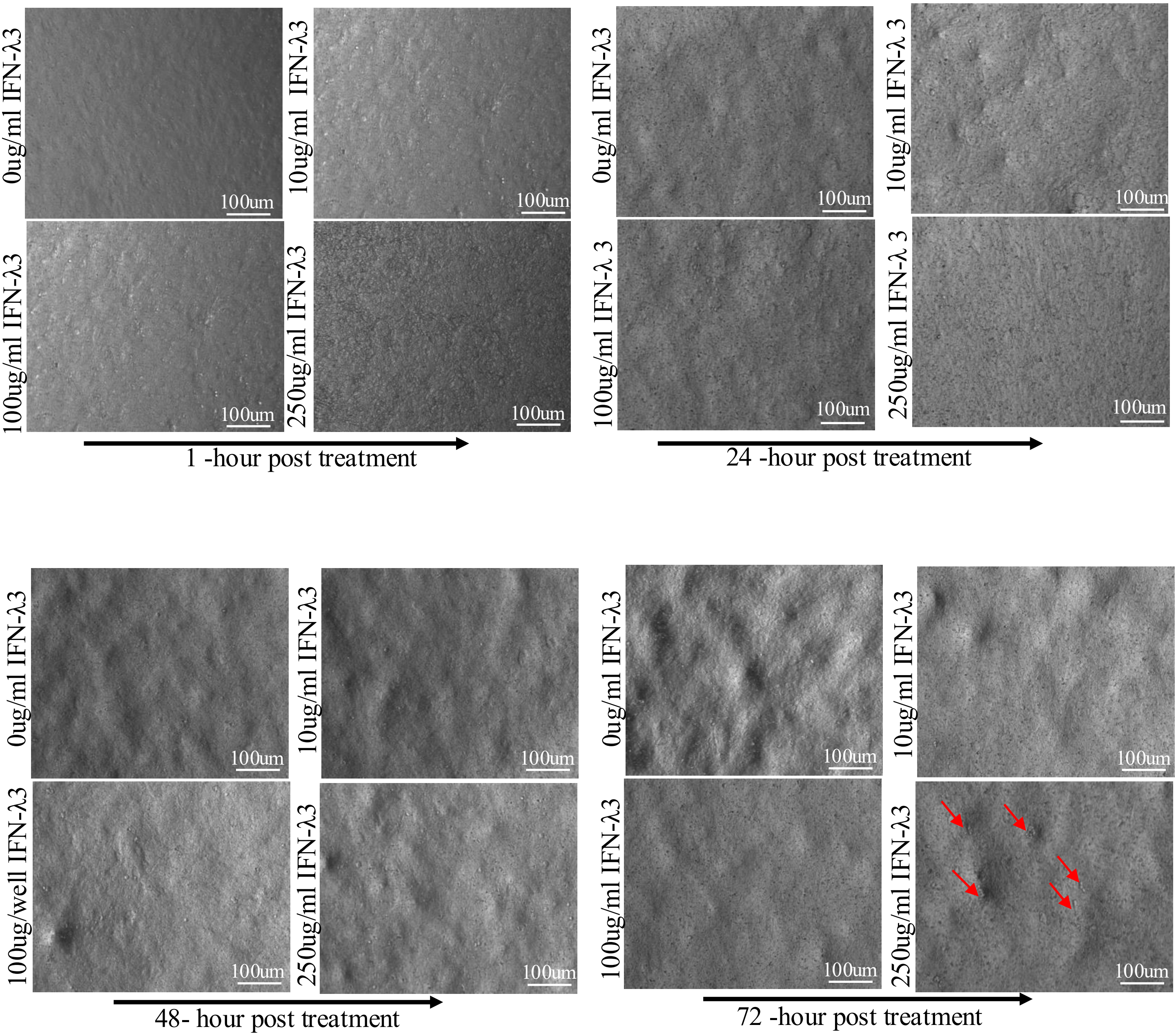
0ug/ml IFN-λ3
10ug/ml IFN-λ3
100ug/ml IFN-λ3
250ug/ml IFN-λ3
100um
1 -hour post treatment
0ug/ml IFN-λ3
10ug/ml IFN-λ 3
100ug/ml IFN-λ 3
250ug/ml IFN-λ 3
24 -hour post treatment
0ug/ml IFN-λ3
10ug/ml IFN-λ3
100ug/well IFN-λ3
250ug/ml IFN-λ3
48- hour post treatment
0ug/ml IFN-λ3
10ug/ml IFN-λ3
100ug/ml IFN-λ3
250ug/ml IFN-λ3
72 -hour post treatment

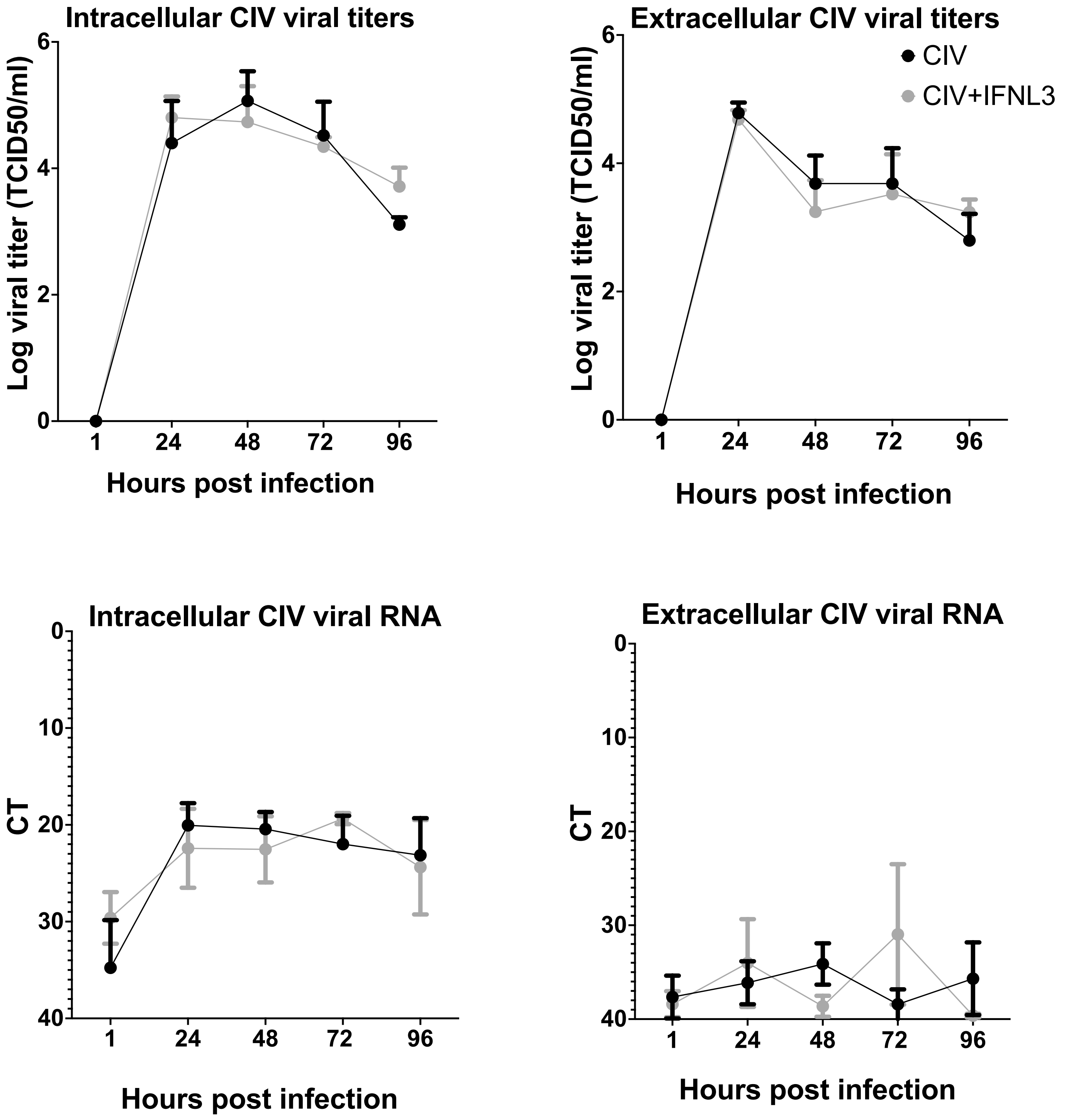

Intracellular CIV viral titers
Log viral titer (TCID50/ml)
Hours post infection
Extracellular CIV viral titers
CIV
CIV+IFNL3
Log viral titer (TCID50/ml)
Hours post infection
Intracellular CIV viral RNA
CT
Hours post infection
Extracellular CIV viral RNA
CT
Hours post infection
